# Gut microbiota reshapes host energy metabolism to modulate depressive behaviors

**DOI:** 10.1080/19490976.2026.2662556

**Published:** 2026-04-23

**Authors:** Pu Lei, Zhiyang Qi, Qingyan Ma, Binbin Zhao, Binglong Wen, Wenhui Jiang, Wenyu Xi, Yixin Liu, Yufeng Xun, Shuo Zhang, Yunpeng Wang, Yijie Guo, Wei Wang, Xiancang Ma, Min Jia, Yajuan Fan

**Affiliations:** a Department of Psychiatry, The First Affiliated Hospital of Xi'an Jiaotong University, Xi'an, China; b Shaanxi Belt and Road Joint Laboratory of Precision Medicine in Psychiatry, The First Affiliated Hospital of Xi'an Jiaotong University, Xi'an, China; c Med-X Institute, Center for Immunological and Metabolic Diseases, The First Affiliated Hospital of Xi’an Jiaotong University, Xi'an, China; d Center for Brain Science, The First Affiliated Hospital of Xi'an Jiaotong University, Xi'an, China

**Keywords:** Depressive disorder, energy metabolism, gut microbiota, TCA/ornithine cycle, glycolysis, fecal microbiota transplantation

## Abstract

Disturbances in energy metabolism are a key pathophysiological feature of major depressive disorder (MDD). The gut microbiota, as a critical regulator of host metabolism, may influence systemic energy homeostasis and contribute to depression. To investigate this, we performed a multi-omics analysis integrating targeted metabolomics and shotgun metagenomics on samples from 100 MDD patients and 68 healthy controls. MDD patients exhibited significant disruptions in central energy pathways (glycolysis, TCA cycle, and ornithine cycle), which correlated with symptom severity and cognitive impairment. We identified 36 bacterial species whose abundances were linked to mitochondrial fatty acid synthesis, ketogenesis, and amino acid metabolism, and were associated with altered levels of core metabolites like lactate and L-glutamic acid. Mediation analysis established a “gut microbiota–energy metabolites–depressive phenotype” axis, where metabolites mediated the effects of specific bacteria (e.g., *Dorea_formicigenerans*) on symptoms. To validate causality, we used a chronic social defeat stress mouse model with simultaneous autologous fecal microbiota transplantation (FMT). FMT effectively reshaped the gut microbiota, ameliorated depression-like behaviors, and reversed the stress-induced shift toward anaerobic glycolysis in serum and the central nervous system. Critically, FMT restored mitochondrial morphology and structural integrity in the prefrontal cortex and hippocampus, renormalizing the relationship between metabolism and behavior. Our findings elucidate the gut microbiota's role in MDD pathogenesis via host energy metabolism regulation and posit early autologous FMT as a novel strategy to correct central energy imbalances.

## Graphical abstract

**Figure:**
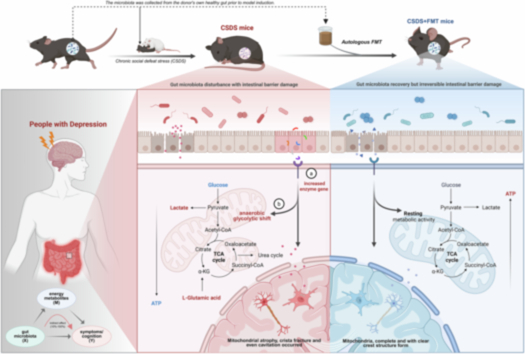

## Introduction

Major depressive disorder (MDD) is a highly disabling psychiatric condition with a substantial global burden, exhibiting a lifetime prevalence of 10%–20%.^1^ Beyond core symptoms such as affective disturbances and cognitive impairment,^2^ accumulating evidence indicates that MDD is accompanied by marked dysregulation of energy metabolism abnormalities, including disruptions in glucose homeostasis, mitochondrial dysfunction, and alterations in multiple metabolic pathways.^3^ Notably, these metabolic disturbances are not merely secondary epiphenomena, also may actively contribute to the development of depression-like behavioral phenotypes and influence clinical outcomes.

The mitochondrial tricarboxylic acid (TCA) cycle constitutes the center of glucose, fatty acid, and amino acid metabolism to produce energy,^4^ and its abnormality can lead to insufficient ATP synthesis and accumulation of reactive oxygen species, which directly affect synaptic plasticity and hippocampal neurogenesis.^5^ Moreover, dysregulation of the ornithine cycle may contribute significantly to cognitive and motor impairment by perturbing urea metabolism and neurotransmitter precursor synthesis.^6^ Increased levels of arginine, a direct precursor of nitric oxide, in patients with MDD, can potentiate oxidative stress in the central nervous system.^7^ As a crucial mechanism in the regulation of excitatory neurotransmitters, abnormal glutamate metabolism (e.g., an increase in glutamate concentration in the synaptic gap) causes neurotoxicity and aggravates the disturbance of energy metabolism by inhibiting the activity of major enzymes in the TCA cycle, thereby forming a vicious cycle.^8^

In recent years, gut microbiota dysbiosis has been consistently reported in patients with MDD.^9^ Such dysbiosis can disrupt cerebral energy supply and mitochondrial function either through the direct secretion of neuroactive metabolites (e.g., glutamate) or by indirectly modulating host metabolic enzyme activity.^10^^,^^11^ High-resolution 16S sequencing analyses have identified significant alterations in the abundance of amino acid-metabolizing bacteria, particularly *Faecalibacterium prausnitzii* and *Escherichia coli*, in the gut microbiota of patients.^12^ These microbial populations may have indirect effects on metabolic flux through the TCA and ornithine cycles by processing crucial precursor molecules such as glutamate and arginine.^13^^,^^14^ Animal evidences further reveal that alterations in the abundance of gut bacteria may directly influence central nervous system energy homeostasis by regulating fatty acid oxidation and ketogenesis.^15^^,^^16^ Nevertheless, current research remains largely correlative, and the mediating role in shaping behavioral phenotypes between gut microbiota and energy metabolism disturbances still lacks systematic validation. Furthermore, although clinical intervention studies have shown that regulating the gut microbiota has certain potential in improving depressive symptoms,^17^^,^^18^ there is still a lack of direct experimental evidence to verify the causal relationship of how these interventions reverse behavioral phenotypes by reshaping energy metabolism pathways. In particular, there is a notable lack of systematic investigation into whether fecal microbiota transplantation (FMT), especially autologous FMT administered during early disease stages, can effectively correct energy metabolism imbalances and associated brain structural and functional abnormalities in MDD models.

To address these research gaps, this study collected serum and fecal samples from 100 MDD patients and 68 age- and sex-matched healthy controls (HCs). By integrating targeted metabolomics with shotgun metagenomic sequencing, we performed multi-omics analyses to identify specific disruptions in energy pathways in MDD from the perspectives of metabolite abundance and microbial functional annotation. Mediation analysis was further applied to elucidate the causal role and specific regulatory networks through which gut microbiota influence depressive phenotypes via energy metabolism mechanisms. Subsequently, using a chronic social defeat stress (CSDS) model to induce depression-like behaviors in mice, we implemented autologous healthy FMT concurrently to monitor its effects on behavioral phenotypes, gut microbiota restructuring, and energy metabolism profiles in both serum and brain tissues.

## Methods

### Study population

One hundred hospitalized patients with depression were recruited from the Department of Psychiatry at the First Affiliated Hospital of Xi'an Jiaotong University between January and December 2023. All patients met the following inclusion criteria: (1) diagnosed with depression according to the Diagnostic and Statistical Manual of Mental Disorders, 5th edition (DSM-V); (2) 17-item Hamilton Depression Rating Scale (HAMD-17) score > 17; (3) aged 18–50 y; (4) completed at least junior high school education; (5) had no history of antidepressant use within the six months prior to enrollment, and had taken antidepressants for less than three days at the time of enrollment; (6) no significant history of infection or use of anti-inflammatory drugs in the past month; and (7) no obvious physical diseases. Among the 100 enrolled patients, 47 were treatment-naïve, while the remaining 53 had prior antidepressant exposure with a mean treatment duration of 3.4 months.

During this period, 68 age- and sex-matched HCs were recruited through poster recruitment. None of the HCs had any psychiatric disorders diagnosed by the DSM-V and had no history of anti-inflammatory drug use or obvious physical disorders. This study adhered to all principles and regulations of the Declaration of Helsinki and was approved by the Ethics Committee of the First Affiliated Hospital of Xi'an Jiaotong University (No. XJTU1AF2022LSK-249). All the participants were aware of the study content and provided written informed consent. Furthermore, this study was registered on the clinical trials with the number NCT06100302.

### Clinical and cognition evaluation

On the day of enrollment, a case report form was used to collect general data and information on bowel habits from the participants. Depression and anxiety were evaluated using the Patient Health Questionnaire-9 (PHQ-9) and Anxiety Disorder Scale-7 (GAD-7) self-rating scales, respectively. Two professional psychiatrists with consistent training used the HAMD-17 and Hamilton Anxiety Rating Scale (HAMA) to evaluate the patients' depression and anxiety. The cognitive function of the participants was evaluated using the THINC Integrated Tool (THINC-IT)^19^ within three days of enrollment. The tool consists of four objective tests that primarily assess attention, working memory, and executive function, such as the Choice Reaction Time (CRT), 1-Back Memory Task (1-Back), Digit Symbol Substitution Test (DSST), and Trail Making Test-Part B (TMT-B), as well as self-perceived cognitive deficits, and the Perceived Deficits Questionnaire for Depression (PDQ-5-D), which measures attention, organizational, retrospective, and prospective memory performance. To make the results easier to understand, we multiplied the test data of CRT, TMT-B, and PDQ-5-D by -one during statistical analysis. The higher the score on the five THINC-it tests, the better the cognitive function.

### Collection of serum and fecal samples

On the second day of enrollment, 2 ml of fasting venous blood was collected in an anticoagulant tube to determine the number of blood cells (leukocytes, neutrophils, lymphocytes, and monocytes) and hemoglobin (HGB) content. Five ml venous blood was collected in coagulation tubes. After centrifugation, a portion of the serum was used to measure lipid levels (triglycerides, cholesterol [CHO], low-density lipoprotein [LDL], and high-density lipoprotein [HDL]) and thyroid hormones (free triiodothyronine [T3], free thyroxine [FT4], and thyroid stimulating hormone [TSH]). The other portion was quickly frozen in liquid nitrogen and stored at −80 °C for the detection of energy metabolites. Feces were collected using a sterile stool collector within three days of inclusion, and DNA was extracted using the Magbeads Fast DNA^®^ kit (MP Biomedicals, Shanghai, China) and stored at −80 °C for metagenomic sequencing.

### Measurement and analysis of energy metabolites

Ultra-high performance liquid chromatography (UPLC) (Waters ACQUITY H-Class) coupled with tandem mass spectrometry was employed to quantitatively profile 68 energy-related metabolites. Among these, a total of 35 metabolites were successfully identified in human serum (Table S1). Prior to statistical analysis, raw peak areas were normalized using PQN (probabilistic quotient normalization) and log‑transformed to improve normality. The metabolome data were analyzed according to the orthogonal partial least squares discriminant analysis (OPLS-DA) model (R, ropls package), and score charts of each group were drawn to show the differences between the groups. Differential metabolites were screened based on variable importance projection (VIP) >1 from the OPLS-DA module and a *p*-value < 0.05 from univariate analysis.

### Metagenomic sequencing and analysis

The concentration and integrity of the DNA were detected using Qubit and agarose gel electrophoresis, respectively. Qualified DNA samples were used for library development and detection, and MGISEQ-T7 PE150bp was used for sequencing. Fastp and Bowtie2 software were used to control the quality of the sequencing data and filter out readings that might have originated from the host, respectively. Metagenomic sequencing data were annotated using MetaPhlAn4^20^^,^^21^ software, and the microbial community composition was analyzed against metagenomic data to obtain relative abundance information. The alpha and beta diversity of the microbiota were assessed using the Shannon index and Principal Coordinate Analysis (PCoA) based on Bray–Curtis distances, respectively, implemented with the vegan package in R. Permutational multivariate analysis of variance (PERMANOVA) was performed using the adonis2 function to evaluate the contribution of factors (such as group, PHQ etc.) to microbial community differences, with statistical significance determined by permutation tests. Differential abundance between groups was examined using the Wilcoxon rank-sum test. To identify taxa with significant differential abundance and estimate their effect sizes, linear discriminant analysis effect size (LEfSe) was conducted following the standard Galaxy workflow. All *p*-values from multiple comparisons were adjusted using the Benjamini–Hochberg false discovery rate (FDR) correction. Bowtie2^22^ and gut metabolic modules (GMM)^23^ were used to identify and analyze metagenomic data to obtain a GMM related to energy metabolism. Furthermore, HUMAnN3^21^^,^^24^ was used to identify species-level functional composition and enzymes of known species in a population over a tiered retrieval strategy, thereby achieving quantitative gene family and pathway abundance as well as rapid access to diverse species and functional compositions. Samples with insufficient DNA quantity or quality, failure to pass sequencing quality control, or incomplete sequencing data were excluded. As a result, metagenomic sequencing was performed on samples from 76 MDD patients and 53 HCs. The metagenomic sequencing data reported in this paper can be found in the Genome Sequence Archive database (CRA019246).

### CSDS model and FMT

A total of 36 6–7-week-old male C57BL/6 mice were purchased from Beijing Vital River Laboratory Animal Technology Co., Ltd. After a one-week acclimatization, the C57BL/6 mice were randomly assigned to either a control (CON) group or a CSDS group, with an allocation ratio of 1:2 (CON: CSDS). Duration the 14-d CSDS procedure, the defeated mice were randomized into two groups in a 1:1 ratio. One group (CSDS+FMT) received the prepared bacterial suspension (approximately 0.2–0.3 mL) via oral gavage 6 h after each daily defeat session. These mice underwent autologous FMT via individual-matched transplantation of the fecal bacterial suspension prepared from their own pre-modeling (acclimation period) samples. Fecal samples were collected prior to any CSDS exposure, while all mice were behaviorally naïve. The other CSDS group and the CON group received an equal volume of saline via gavage. Behavior assessments (SPT, OFT, TST, and NOR) were conducted following the completion of the modeling period.

### Electron microscopy sample observation

The morphology and quantity of mitochondria in microglia and neurons in PFC and HIP were observed by electron microscopy. Blocks were trimmed to a pyramidal tip (~1 × 1 mm) under a stereomicroscope. Ultrathin sections (~90 nm) were cut using an ultramicrotome and collected on copper grids. Grids were examined using a transmission electron microscope. Target cells were located at low magnification (2,000–5,000X). High-magnification images were acquired (15,000–30,000X for overall mitochondrial morphology; >50,000X for cristae details).

### Statistical analyses

All statistical analyses were performed using SPSS25 and R4.2.2. Continuous variables were statistically analyzed using two independent sample t-tests (normally distributed) or non-parametric tests (non-normally distributed). Categorical variables were represented as n(%) and analyzed using the chi-square test. Analysis of covariance was used to compare cognitive test results between the groups. Adonis was used to analyze the explanations of clinical biochemical indicators and symptom scales for microbial differences (vegan package), and significance was verified using a permutation test. The relationship between energy metabolite levels, gut microbiota, and cognition was investigated using parallel mediation analysis^25^ (mediation package).

For comparisons in animal experiments (including behavior tests, intestinal permeability protein expression, and mitochondrial parameters) across groups, one-way analysis of variance (ANOVA) was used to assess overall statistical differences. If a significant main effect was detected, Tukey's honest significant difference (HSD) post hoc test was applied for pairwise comparisons to control for type I error inflation. Data in statistical graphs are presented as the mean ± standard error of the mean (SEM). Fold change (FC) was calculated to evaluate the relative differences in energy metabolite levels between groups. Pearson correlation analysis was performed to examine linear relationships between behavioral phenotypes and energy metabolites, as well as between behavioral phenotypes and mitochondrial metrics (stats, Hmis, and corrplot packages). Additionally, co-inertia analysis (CIA) was employed to explore the global association structure between multiple energy metabolites and multidimensional behavioral phenotypes (ade4 package). This multivariate method quantifies the co-variation between two datasets, such as metabolite profiles and behavior measurements, revealing underlying integrative relationships.

## Results

### Comparison of demographic characteristics and cognitive function between two groups

**Figure 1.**
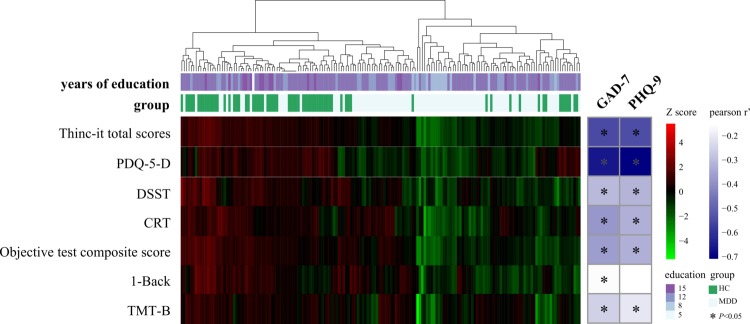
Expression of cognitive differences between groups and their association with symptoms. On the left, the heatmap illustrates that patients with major depressive disorder (MDD) performed significantly worse than healthy controls (HC) across multiple subjective and objective cognitive assessments, including PDQ-5-D, 1-Back, DSST, CRT, TMT-B, objective test composite score, and the Thinc-it total score. Red indicates higher cognitive function (positive *Z*-score), while green indicates lower cognitive function (negative *Z*-score). On the right, the heatmap displays Pearson partial correlation coefficients (adjusted for years of education) between depression/anxiety symptom scores and composite objective cognitive performance. Darker colors represent stronger correlations. *p < 0.05.

The study included 100 patients with depression and 68 HCs. In addition to years of education, no significant differences were observed in sex, age, BMI, smoking, drinking, blood pressure, abdominal circumference, or hip circumference between the two groups. Compared with HCs, individuals with depression had a lower daily defecation frequency and harder fecal properties, and showed lower levels of blood lipids (CHO, LDL, and HDL), HGB, percentage of neutrophils, and higher percentages of lymphocytes and monocytes (Table 1). This indicates that the nutritional status of patients with depression is poor, and their immune function is abnormal. Pearson’s analysis showed a significant positive correlation between depression and anxiety symptoms in patients and an obvious consistency between the self-rated and examiner-rated scales (Figure S1A). After adjusting for years of education, patients with depression showed significantly impaired overall and subdomain cognitive function, which correlated negatively with the severity of depression and anxiety symptoms (Figure 1). A particularly strong association was observed between the subjective cognitive measure (PDQ-5-D) and symptom scales. Importantly, when we further adjusted for additional variables (*p* < 0.05) that differed between groups in Table 1, the significant negative correlation between depression severity and cognitive performance remained essentially unchanged, supporting the robustness of the primary finding (Figure S1B).

**Table 1.** Comparison of general characteristics and clinical data between groups.

	HC group (*n* = 68)	MDD group (*n* = 100)	t/χ^2^	*p*-value
Age, years	27.25 ± 5.33	25.60 ± 8.10	1.579	0.116
Male, *n* (%)	26 (38.2)	30 (30.0)	1.235	0.266
BMI, kg/m²	23.14 ± 3.37	22.00 ± 4.76	1.822	0.07
Education, years	15.82 ± 2.57	14.20 ± 2.80	3.879	<0.001
SBP	112.28 ± 11.39	110.17 ± 14.49	1.007	0.315
DBP	75.62 ± 9.16	76.26 ± 9.66	-0.432	0.666
Abdominal girth	79.75 ± 10.42	79.81 ± 11.58	-0.034	0.973
Hip circumference	94.85 ± 7.62	94.44 ± 8.65	0.318	0.751
Smoking, *n* (%)	11 (16.2)	17 (17.0)	0.020	0.888
Alcohol, *n* (%)	14 (20.6)	24 (24.0)	0.269	0.604
Daily defecation frequency, *n* (%)			22.513	<0.001
More than twice	4 (5.9)	4 (4.0)		
Once or twice	52 (76.5)	42 (42.0)		
Once every two days	12 (17.6)	54 (54.0)		
Fecal property, *n* (%)			20.798	<0.001
Soft	62 (91.2)	59 (59.0)		
Hard	6 (8.8)	41 (41.0)		
WBC	5.75 ± 1.40	6.07 ± 1.54	−1.415	0.159
Neutrophil%	58.71 ± 6.76	54.83 ± 9.66	3.054	0.003
Lymphocyte%	33.04 ± 6.93	36.93 ± 8.32	−3.175	0.002
Monocyte%	5.62 ± 1.17	6.91 ± 1.94	−5.344	<0.001
HGB	143.78 ± 17.47	131.91 ± 17.19	4.364	<0.001
TG	1.21 ± 0.79	1.06 ± 0.57	1.357	0.178
HDL	1.35 ± 0.27	1.10 ± 0.27	5.909	<0.001
LDL	2.65 ± 0.69	2.34 ± 0.67	2.859	0.005
CHO	4.52 ± 0.72	3.89 ± 0.77	5.358	<0.001
PHQ-9	3.24 ± 3.86	18.88 ± 5.65	−21.318	<0.001
GAD-7	2.01 ± 2.67	12.01 ± 5.64	−15.372	<0.001
Duration of disease, months		27.43 ± 28.43		
HAMD-17		25.02 ± 5.02		
HAMA		21.65 ± 7.88		

### Abnormal energy metabolism in patients with depression

**Figure 2.**
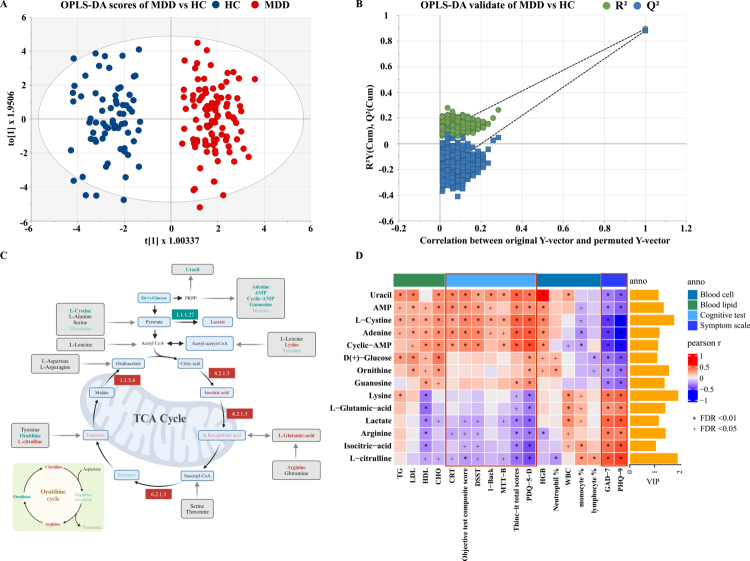
Patients with MDD exhibited significant disturbances in energy metabolism processes. (A) OPLS-DA score plots demonstrated that energy metabolites could effectively discriminate between MDD and HCs, with the model exhibiting robust stability (B). (C) Significant alterations were observed in metabolites associated with the TCA cycle, ornithine cycle, and various energy metabolism-related pathways (including amino acids, purines, and pyrimidines). Metabolites highlighted in red and green denote significantly elevated or reduced levels in the MDD group, respectively. The bold metabolites indicate compounds with VIP scores >1 and statistically significant differences (*p*-value < 0.05) in the OPLS-DA model. Similarly, red and green boxes indicate significantly upregulated or downregulated enzyme activities in MDD patients. (D) The perturbed energy metabolites in MDD showed significant correlations with serum biochemical markers, cognitive performance, and clinical symptom severity.

Thirty-five energy-related metabolites were detected using targeted liquid chromatography-tandem mass spectrometry. The OPLS-DA score plot showed that these energy metabolites significantly separated the MDD group from the HC group, and the permutation test confirmed that the model was stable and reliable (Figure 2A and B). Twenty-two different metabolites were initially selected by univariate analysis with a *p*-value < 0.05, whereas 14 different energy metabolites were identified by combining the analysis with VIP values > 1 in OPLS-DA (Table S1A). The levels of glucose, L-cysteine, ornithine, guanosine, uracil, adenine, cyclic-AMP, and AMP in the serum were significantly lower in the MDD group, whereas the levels of L-citrulline, lysine, arginine, L-glutamic acid, lactate, and isocitric acid were significantly higher in the MDD group. Moreover, these energy metabolites were correlated with each other, especially adenine and cyclic AMP, which were significantly correlated with all metabolites (Figure S2). To further rule out potential confounding by prior antidepressant use, we compared the energy metabolites between the two medication-history subgroups (treatment-naïve, *n* = 47; prior antidepressant use, *n* = 53). No significant differences were observed for any of these metabolites (Table S1B).

Generally, MDD is often accompanied by decreased appetite in addition to depressive mood and cognitive impairment, and some patients may exhibit weight loss. Although the patient did not show a significantly low BMI, decreased blood glucose levels and abnormal amino acid metabolism were observed, which affected the energy metabolism process of the patient. For example, it was found that patients have significantly lower levels of purine and pyrimidine metabolites, which have been shown to be involved in communication between neurons, astrocytes, and microglia.^26^ In addition to the TCA cycle, the ornithine cycle was significantly altered, resulting in significantly lower ornithine and higher arginine levels (Figure 2C). Arginine is a precursor of several signaling molecules that can accelerate cognitive impairment by promoting elevated glutamate levels, leading to excitotoxicity and neuronal cell death. Interestingly, these energy metabolites, particularly those in the purine, pyrimidine, and ornithine cycles, were significantly associated with cognitive function tests, anxiety, and depressive symptoms in the entire cohort. Energy metabolites were positively correlated with subjective and total cognitive test scores than objective cognitive tests. Moreover, metabolites that were significantly decreased in patients were positively correlated with blood lipid and HGB levels, whereas those that were significantly increased in patients were positively correlated with the proportion of immune cells (Figure 2D). Notably, a stronger association between energy metabolites and thyroid hormone levels (including thyroid-stimulating hormone, T3, and FT4) was observed in the MDD group (Figure S3), suggesting a significant relationship between abnormal energy metabolism and hypothyroidism in patients with depression.

### Gut microbiota disturbance and its association with energy metabolites

**Figure 3.**
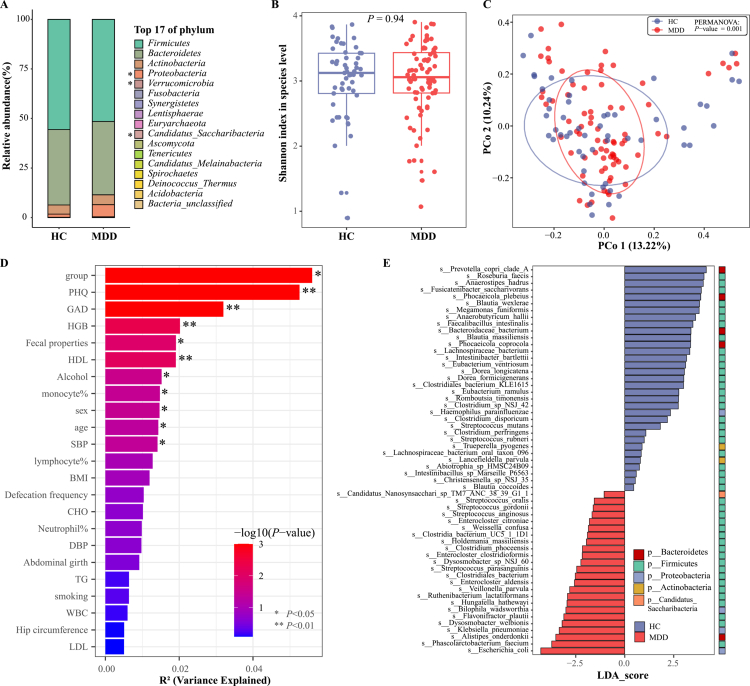
Patients with MDD exhibited significant alterations in gut microbiota composition. (A) Phylum-level taxonomic profiles and intergroup differences between MDD and HCs, displaying the top 17 bacterial phyla. (B) While no significant differences were observed in α-diversity (Shannon index), β-diversity (PCoA analysis) revealed distinct clustering patterns between MDD patients and HCs (C). (D) Multivariate analysis of variance (Adonis) identified variables significantly influencing gut microbial community structure. (E) Linear discriminant analysis (LDA) effect size (LEfSe) analysis highlighted species-level taxonomic features that discriminated between groups.

Since the human gut microbiota regulates the processing of ingested food and metabolic processes of energy and nutrients in the human body, the same food can have different effects on human energy metabolism in different gut microbiota environments. We used metagenomic sequencing to analyze structural differences in the gut microbiota of 76 patients with MDD and 53 HCs, and annotated the energy metabolic potential of the different bacteria between the groups. At the phylum level, we identified 17 bacterial phyla, among which three (Proteobacteria, Candidatus Saccharibacteria, and Verrucomicrobia) were significantly enriched in the MDD group compared to HCs (Figure 3A). These bacteria have been reported to be significantly increased in patients with MDD and mouse models in previous studies.^27^^,^^28^ At the species level, no significant change in *α*-diversity was observed in patients with depression, whereas *β*-diversity showed a significant difference compared to the HC group after conducting a PERMANOVA test (Figure 3B and C). Multivariate analysis of variance revealed that group assignment, depressive symptoms, and anxiety symptoms were the strongest explanatory variables for bacterial species variation, accounting for 5.6%, 5.3%, and 3.2% of the variance, respectively. Secondary contributors including hemoglobin levels, fecal properties, high-density lipoprotein cholesterol, alcohol consumption, monocyte percentage, sex, age, and systolic blood pressure individually explained 1.4% to 2.0% of the observed variance. The remaining indicators such as lymphocyte percentage, body mass index, smoking status, and white blood cell count demonstrated no significant explanatory power for the microbial community variation (Figure 3D). After removing less than 5% of the bacterial species from the samples, we found that 92 species were significantly altered in the MDD group using non-parametric tests, of which 50 and 42 species were significantly high and low, respectively (Table S2). Notably, LDA was used to further estimate the influence of each bacterial abundance on the difference effect, revealing that 121 bacteria showed significant changes from the phylum to the species level, including 57 different species, of which almost 80% belonged to Firmicutes (Table S3 and Figure 3E).

**Figure 4.**
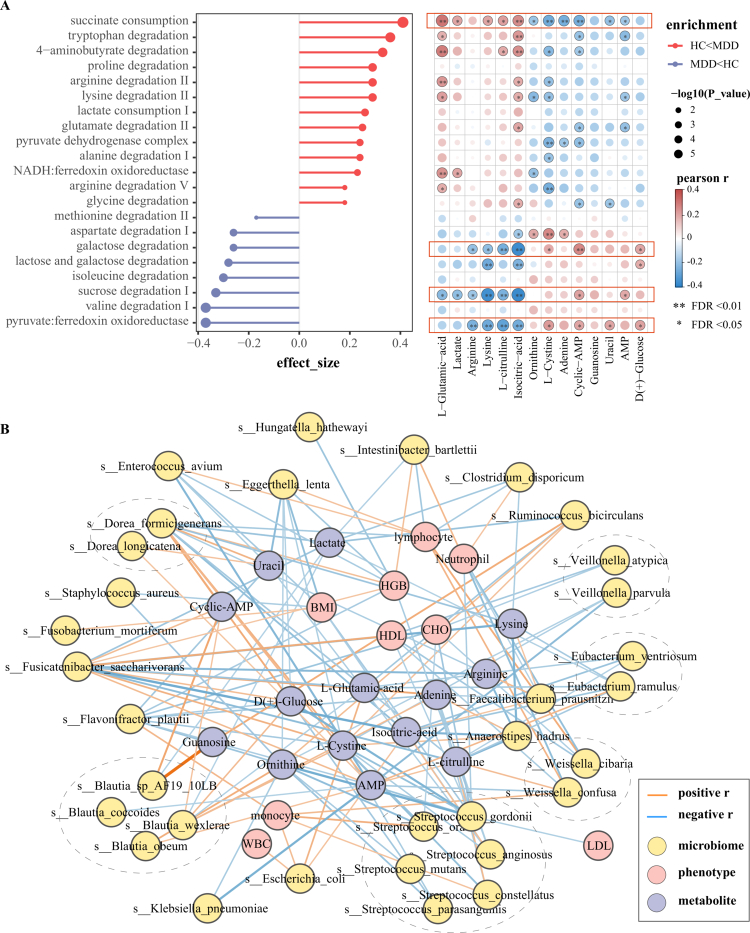
Gut microbial functions and species associated with energy metabolism pathways. (A) Gut metagenomic analysis (GMM) identified 21 microbial metabolic pathways significantly associated with host energy metabolism. These pathways demonstrated robust correlations with key energy-related metabolites. (B) Significant correlations among thirty-six gut bacterial species significantly involved in energy metabolism pathways, phenotypic indicators, and metabolites (only results with *p* < 0.05 from Spearman correlation analysis are displayed). Nodes are color-coded by type: yellow for microorganisms (labeled with specific species names), pink for phenotypic indicators (e.g., BMI, WBC), and purples for metabolites (e.g., lactate, uracil). Some microbial species are highlighted with dashed borders, indicating their affiliation with taxonomically clustered groups. Edge colors denote the direction of correlation: orange for positive (*r* > 0) and blue for negative (*r* < 0).

Using the GMM to predict the functional potential of the gut microbiota, we identified 33 functional modules (Table S4), of which 21 were related to human energy metabolism (Figure 4A). Succinate/lactate consumption, tryptophan degradation, and proline/arginine/lysine degradation were significantly increased in the MDD group, whereas lactose, galactose, and sucrose degradation were significantly reduced. Succinate consumption in the TCA cycle, sucrose degradation, as well as lactose and galactose degradation were significantly correlated with the levels of amino acids in the serum, such as L-glutamic acid, L-citrulline, lysine, and L-cysteine. This emphasizes that the human gut microbiota affects energy metabolism from the perspective of amino acid metabolism. Furthermore, we used HUMAnN3 analysis to obtain information regarding microbial metabolic mechanisms and functional modules and found that the mechanisms involved in energy metabolism included PWY66−367 (ketogenesis), PWY−7942 (5−oxo−L−proline metabolism), PWY66−430 (myristate biosynthesis, mitochondria), PWY66−429 (fatty acid biosynthesis initiation, mitochondria), and PWY66−409 (the super mechanisms of purine nucleotide salvage), etc (Figure S4). Notably, 36 species participated in the metabolism of PWY66-429 (Figure S5). Among them, *Escherichia_coli* and *Klebsiella_pneumoniae* which were highly concentrated in the MDD group were involved in PWY66-409, PWY66-429, PWY66−430, and PWY−7942 (Table S5). The correlation results showed that bacteria such as *Dorea_formicigenerans*, *Fusicatenibacter_saccharivorans*, *Anaerostipes_hadrus*, *Eubacterium_ramulus* were significantly related to energy metabolites, especially ornithine cycle metabolites, but were less correlated with blood lipids and the proportion of immune cells (Figure 4B). Notably, although some depression-associated microbiota alterations (such *Akkermansia_muciniphila*) did not directly participate in energy metabolism, they also showed significant correlations with energy metabolites (Figure S6), thereby warranting further investigation of the mechanisms underlying these relationships. Moreover, we observed significant alterations in the levels of TCA cycle enzymes encoded by the gut microbiota. The gene abundance of aconitate hydratase (EC4.2.1.3), succinate–CoA ligase (EC6.2.1.5), and malate dehydrogenase (EC1.1.5.4) increased, whereas that of L-lactate dehydrogenase (EC1.1.1.27) significantly decreased (Figure S7A, Table S6). We found 27 significantly differentially abundant bacterial species between groups (such as *Dorea_formicigenerans*, *Fusicatenibacter_saccharivorans*, and *Escherichia_coli*) could effectively express EC1.1.1.27. Furthermore, seven, five and two distinct species exhibited significant expression of EC4.2.1.3, EC6.2.1.5, and EC1.1.5.4, respectively (Table S7). It was worth noting that *Escherichia_coli* and *Klebsiella_pneumoniae*, which are involved in multiple energy metabolic processes, simultaneously encode these four enzymes (Figure S7B and C).

### Mediation analysis results: gut microbiota promotes depression symptom and cognitive impairment by regulating energy metabolites

Regulation of depression symptom and cognitive function by the gut microbiota has been confirmed in most studies,^29^ and these results suggest that energy metabolites may mediate their effects. To delineate the scope of our mediation analysis, we first performed linear regression between all 36 energy metabolism-related bacterial species and all clinical scales to identify significant species–phenotype associations. These predefined associations formed the basis for subsequent mediation analyses aimed at specifically validating the mediating effects of energy metabolites. As summarized in Table S8, we identified significant mediation effects of gut microbiota on depressive symptoms and cognitive function via energy metabolites. The table provides complete statistical data for 346 microbe–metabolite pairs across 8 key outcome measures, including two mood symptom scales (PHQ-9 and GAD-7) and six cognitive function assessments (CRT, DSST, Objective test composite score, PDQ-5-D, and THINC-it total scores).

**Figure 5.**
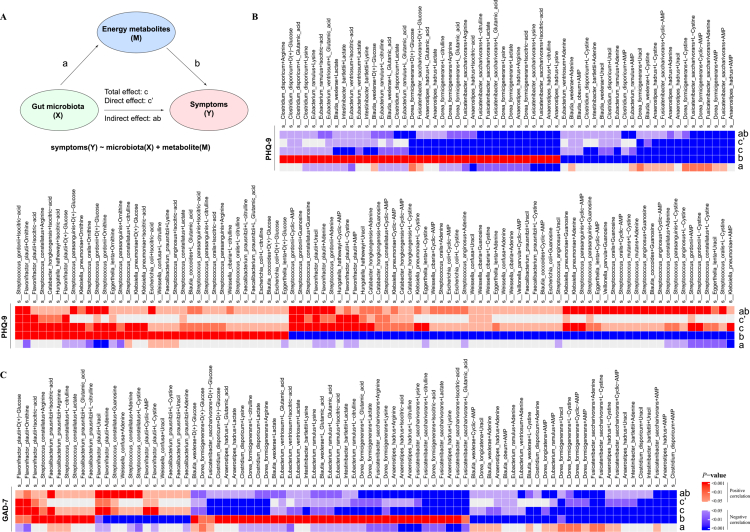
Energy metabolites mediate the regulatory effect of gut microbiota on depressive/anxiety symptoms. (A) schematic model illustrating the mediation of gut microbiota–symptom relationships by energy metabolites. Path a: association between gut microbial species and energy metabolites. Path b: association between energy metabolites and symptoms. Total effect c: overall gut microbiota-symptom relationship. Direct effect c': Microbiota-symptom link independent of metabolites. Indirect effect ab: mediated pathway through energy metabolites. (B) Mediation relationships between gut microbiota and PHQ-9 scores by energy metabolites (152 mediation pairs, *P*_*indirect effect*_ < 0.05). (C) Mediation relationships between gut microbiota and GAD-7 scores by energy metabolites (83 mediation pairs, *P*_*indirect effect*_ < 0.05). Heatmaps display all significant mediation pathways. Statistical significance (*p* < 0.05, *p* < 0.01, and *p* < 0.001) and correlation direction (red: positive correlation; blue: negative correlation) are indicated for each path.

In terms of outcome distribution, the largest number of significant mediation pairs was observed for depressive symptoms (PHQ-9, 152 pairs), followed by anxiety symptoms (GAD-7, 83 pairs) and subjective cognitive measures (PDQ-5-D, 45 pairs). Objective cognitive and composite scores involved 4~31 mediation pairs, suggesting that the gut microbiota–energy metabolite pathway may exert a broader influence on mood symptoms than on cognitive function (Figures 5A–C, S8A–F). The mediating roles of energy metabolites exhibited clear functional specificity. L-cystine (37 pairs, average mediation proportion = 0.506) and adenine (37 pairs, average mediation proportion = 0.439) were the most efficient mediators, broadly involved in both cognitive and mood-related pathways. L-citrulline (30 pairs, average mediation proportion = 0.382) and lactate (29 pairs, average mediation proportion = 0.357) also demonstrated pleiotropic effects across cognitive–mood domains, highlighting their roles as core signaling molecules in the microbiota–neuropsychiatric network. L-glutamic acid (23 pairs, average mediation proportion = 0.255) was primarily associated with mood symptom pathways, with predominantly negative indirect effect estimates, suggesting that its reduction may be linked to mood improvement.

Among the 36 bacterial species examined, 28 showed significant mediation effects, with 23 species participating in ≥3 mediation pairs and only 5 species (e.g., *Blautia_obeum*, *Dorea_longicatena*) involved in fewer than 3 pairs. This indicates that the majority of these microbiota play stable mediating roles within the “microbiota–energy metabolite–neuropsychiatric outcome” pathway. *Dorea_formicigenerans* (52 pairs), *Fusicatenibacter_saccharivorans* (49 pairs), and *Anaerostipes_hadrus* (30 pairs) were the three most frequently implicated species and exhibited consistent mediation efficiency. *Dorea_formicigenerans* (average mediation proportion = 0.282) showed a prominent role in cognitive regulation via cyclic AMP. *Fusicatenibacter_saccharivorans* (average mediation proportion = 0.316) was notably involved in nucleotide metabolite-mediated (e.g., adenine, AMP) cognitive pathways. *Anaerostipes_hadrus* demonstrated a high average mediation proportion (0.361) and was primarily associated with mood symptom regulation through energy metabolites such as lactate, L-glutamic acid, and isocitric acid, with indirect effect estimates ranging from –0.20 to –0.38, suggesting a potential beneficial role in alleviating depressive and anxiety symptoms via downregulation of specific metabolites. Additionally, *Faecalibacterium_prausnitzii* (12 pairs, average mediation proportion = 0.473) and *Blautia_wexlerae* (18 pairs, average mediation proportion = 0.375), though they are involved in fewer mediation pairs, displayed high mediation efficiency, identifying them as potential key functional species in the network.

### Effectively diagnosis of depression based on metabolites associated with cognitive function

To translate the biological insights from our mediation analysis into a clinically applicable tool, we employed a random forest model to screen for diagnostic biomarkers. The model was trained and tuned using all candidate biological indicators from the exploratory mediation analysis (Table S8) within the dedicated training subset (*n* = 90, 70% of the cohort), which included both gut microbiota taxa and energy metabolism-related metabolites. The aim was to determine whether these features could effectively distinguish patients with MDD from HCs.

The feature selection process, optimized to minimize classification error via five-fold cross-validation conducted strictly within the training set, identified a robust and parsimonious optimal subset of seven energy metabolites for diagnostic prediction (Figure S9A). A classifier built exclusively with these seven metabolites demonstrated exceptional performance, achieving areas under the ROC curve (AUC) of 99.95% in the training set and 100% in the completely independent test set (*n* = 39, 30% of the cohort) (Figure S9B and C). Feature importance analysis revealed that isocitric acid contributed the most to the model’s predictive power, followed by L‑citrulline, L‑cysteine, cyclic AMP, adenine, ornithine, and AMP (Figure S9D). Notably, no gut microbiota features were retained in this final parsimonious model, indicating that in our cohort, energy metabolites possessed superior discriminative power for MDD diagnosis compared to microbial taxa alone. Interestingly, these key diagnostic metabolites are known to be primarily produced by gut bacteria such as *Anaerostipes_hadrus*, *Clostridium_disporicum*, *Dorea_formicigenerans*, *Eubacterium_ramulus*, and *Fusicatenibacter_saccharivorans*, hinting at a potential microbial origin for the altered metabolic signature in depression.

### Autologous FMT rescued CSDS-induced depression-like behaviors and normalized the perturbed gut microbiota structure and function

**Figure 6.**
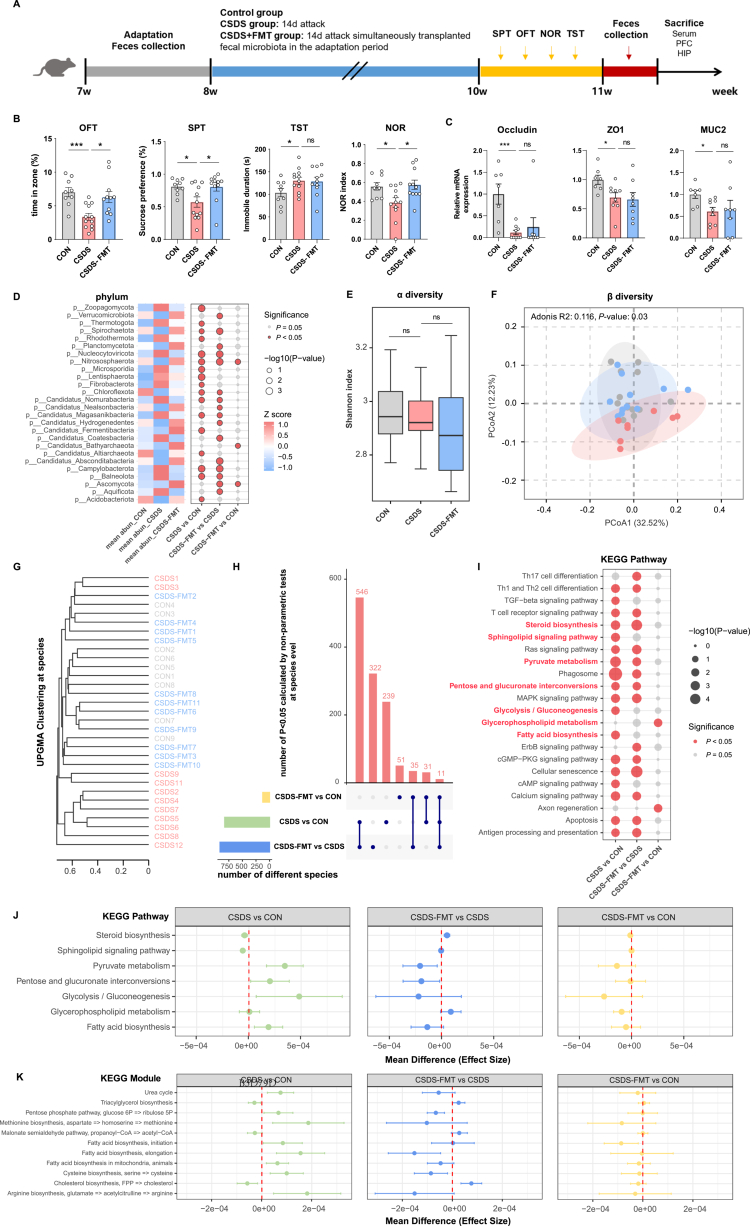
Effects of CSDS and FMT on gut microbiota, intestinal barrier function, and related signaling pathways in mice. (A) Schematic timeline of the experimental design and behavioral tests. (B and C) Data are presented as box plots showing the differences among the three groups in behavior tests (OFT, SPT, TST, and NOR) and the mRNA expression levels of intestinal permeability-related proteins (Occludin, ZO-1, and MUC2). Statistical differences were evaluated by one-way analysis of variance (ANOVA), followed by Tukey's HSD post hoc test for pairwise comparisons if a significant overall difference was detected. *p < 0.05, **p < 0.01, ***p < 0.001. (D) Heatmap showing the mean abundance of significantly differential bacterial phyla across groups (19 phyla). Bubble plot displayed between-group *p*-values (non-parametric test) and significance levels. (E) Shannon index analysis of gut microbiota alpha diversity. (F) Beta diversity visualized by PCoA based on Bray–Curtis distance; group differences were assessed using Adonis. (G) UPGMA clustering tree based on gut microbial species composition, illustrated phylogenetic relationships among all samples (including CON, CSDS, and CSDS-FMT subgroups). CSDS-FMT subgroups showed closer clustering to the CON group and clear separation from the CSDS group. (H) Upset plot showed the number of differential bacterial species across group comparisons. (I) Bubble plot showed significantly enriched KEGG pathways among the three groups, including Th17 cell differentiation, TGF-β signaling pathway, glycolysis/gluconeogenesis, fatty acid biosynthesis, and others. (J) Forest plot presented the mean effect size differences in energy metabolism-related KEGG pathways among the three groups. Positive values indicate pathway activation; negative values indicate pathway inhibition. (K) Forest plot showed effect size differences in KEGG modules related to energy metabolism (11 modules), including arginine biosynthesis, cholesterol biosynthesis, TCA cycle, and others.

To further investigate the role of the gut microbiota in depressive symptoms and energy metabolism dysregulation, we administered fecal microbiota transplantation (FMT) via oral gavage to the model mice during the CSDS procedure, using microbiota collected during the acclimation period (designated as the CSDS-FMT group), with interventions continuing for 14 d (Figure 6A). Control group (CON) and another CSDS model group (CSDS group) received an equal volume of saline via gavage. After modeling, mice in the CSDS group exhibited significant depression-like behaviors, specifically reflected as reduced sucrose preference ratio in the SPT, increased immobility duration in the TST, and decreased time in the zone area in the OFT. Additionally, their memory function was impaired, as indicated by a significantly lower NOR index. In contrast, FMT intervention administered concurrently with CSDS modeling markedly alleviated depression-like behaviors and improved memory function (Figure 6B). Subsequently, CSDS mice were stratified into susceptible (*n* = 9) and resilient (*n* = 3) subgroups based on a composite behavioral score. This analysis revealed that the beneficial effects of concurrent FMT were significantly more pronounced when compared specifically to the CSDS-susceptible subgroup, whereas no significant difference was observed between the FMT group and the CSDS-resilient subgroup (Figure S10). Measurement of mRNA expression levels of gut permeability markers (Occludin, ZO1, and MUC2) revealed significant impairment of intestinal barrier integrity in CSDS mice, which was not restored to normal levels by FMT intervention (Figure 6C).

Metagenomic sequencing analysis demonstrated distinct gut microbial structures between the CON and CSDS groups. At the phylum level, compared with the CON group, the CSDS group showed significant enrichment of 11 phyla (e.g., Balneolota, Campylobacterota, and Fibrobacterota) and reduction in 6 phyla (e.g., Acidobacteriota, Nitrososphaerota, and Spirochaetota). Following FMT, the microbial structure in the CSDS-FMT group shifted toward that of the CON group, with only a few phyla remaining significantly different between groups (Figure 6D, Table S9). Analysis at the species level revealed no significant differences in alpha diversity among the three groups (Figure 6E), whereas beta diversity indicated partial but significant separation in overall community structure, with the CON and CSDS-FMT groups clustering more closely (Figure 6F). UPGMA clustering further showed that CSDS group samples formed a distinct cluster, while CON and CSDS-FMT group samples aggregated together (Figure 6G). Non-parametric testing identified 827 differentially abundant species (*p* < 0.05) between the CSDS and CON groups, 914 between the CSDS-FMT and CSDS groups, but only 128 between the CSDS-FMT and CON groups. Eleven species were consistently differentially abundant across all three groups, including 2 from Campylobacterota, 5 from Bacteroidota, 2 from Pseudomonadota, and 2 from Bacillota (Figure 6H, Table S10). LEfSe analysis (LDA > 2, *p* < 0.05) further identified differentially species between groups. Compared with the CON group, the CSDS group exhibited 38 enriched and 15 depleted species, including *Akkermansia_muciniphila*, a widely studied species, which was significantly reduced in the CSDS group (Figure S11A) but increased significantly after FMT intervention (Figure S11B). Furthermore, only 6 species were significantly enriched in the CSDS-FMT group compared with the CON group (Figure S11C).

Analysis of metabolic pathways associated with differentially abundant microbial species identified 22 KEGG pathways related to MDD, primarily involving metabolic reprogramming, cellular signal transduction, and activation of immune-inflammatory responses. Among these, 7 pathways were closely associated with energy metabolism (Figure 6I). Compared with the CON group, the CSDS group showed significant downregulation of steroid biosynthesis and sphingolipid signaling pathway, while pyruvate metabolism, pentose and glucuronate interconversions, glycolysis/gluconeogenesis, and fatty acid biosynthesis were significantly upregulated. Following FMT intervention, these alterations in energy metabolism pathways were reversed, showing no significant difference from the CON group (Figure 6J). KEGG module analysis further revealed microbial energy metabolism modules associated with MDD. These included M00029 (urea cycle), M00006 (pentose phosphate pathway, oxidative phase, glucose 6P => ribulose 5P), M00017 (methionine biosynthesis, aspartate => homoserine => methionine), M00082 (fatty acid biosynthesis, initiation), M00083 (fatty acid biosynthesis, elongation), M00873 (Fatty acid biosynthesis in mitochondria, animals), and M00021 (cysteine biosynthesis, serine => cysteine), all of which were significantly upregulated in the CSDS group. In contrast, modules M00089 (triacylglycerol biosynthesis), M00013 (malonate semialdehyde pathway, propanoyl-CoA => acetyl-CoA), and M00101 (cholesterol biosynthesis, FPP => cholesterol) were significantly downregulated. After FMT, certain energy metabolism modules such as M00006, M00083, and M00021 exhibited a downward trend (Figure 6K). Furthermore, analysis of energy metabolism-related enzymes (previously listed in Table S6) showed that the levels of EC 6.3.4.5, EC 3.5.3.6, EC 2.3.3.5, and EC 1.1.5.4 were significantly elevated in the CSDS group. FMT intervention significantly reduced the levels of these enzymes, while the level of EC 6.2.1.5 was markedly increased (Figure S11D).

In summary, CSDS mice exhibited depression-like behaviors accompanied by impaired intestinal barrier function and disrupted gut microbiota structure. FMT not only reversed depression-like behaviors and microbial dysbiosis but also corrected associated abnormal energy metabolism pathways, although it did not restore the compromised intestinal barrier integrity.

### Autologous FMT alleviates CSDS-induced energy metabolism imbalance and mitochondrial morphological defects

**Figure 7.**
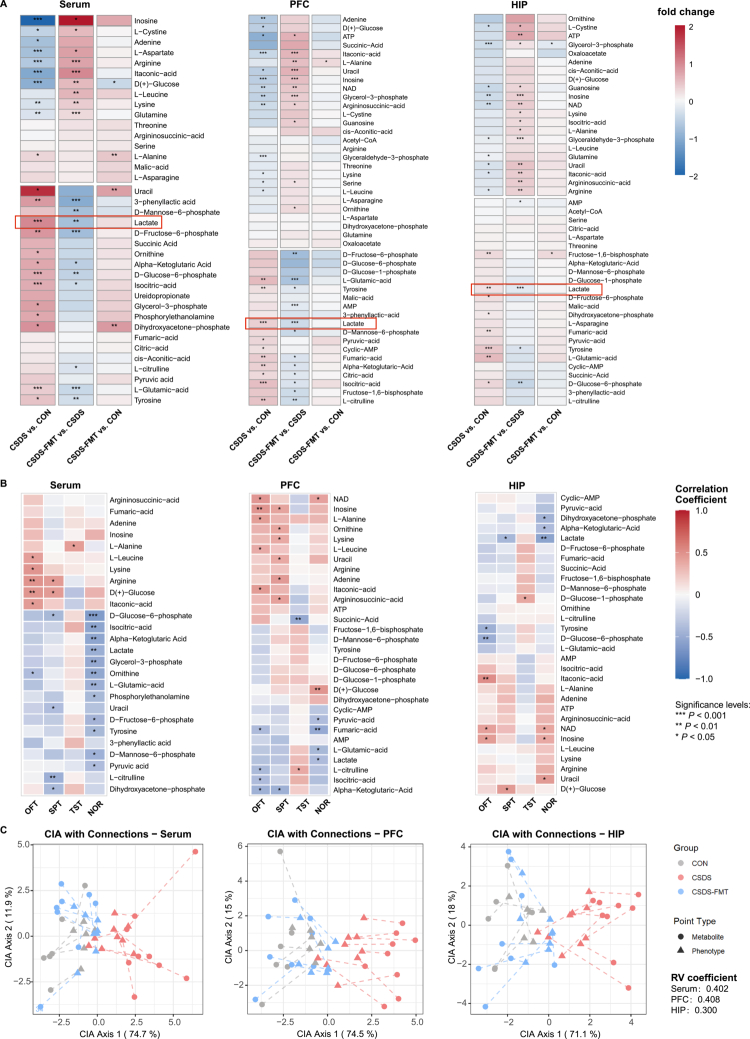
Effects of CSDS and FMT on serum and central energy metabolites in mice. (A) Fold difference in relative expression of more than 40 energy metabolites in serum, PFC, and HIP of the three groups of mice. Metabolite upregulation was indicated in red and downregulation in blue, with color intensity reflecting the magnitude of change. Group comparisons included CSDS vs. CON, CSDS-FMT vs. CSDS, and CSDS-FMT vs. CON. (B) Heatmap of correlations between metabolites in serum, PFC, and HIP and behavioral phenotypes. Color intensity and numerical labels represented the correlation coefficients (ranging from  −1.0 to 1.0), with red indicating positive correlations and blue indicating negative correlations. (C) Co-inertia analysis (CIA) was used to visualize the global relationship between metabolomic profiles (from serum, PFC, and HIP) and behavioral phenotypes. The horizontal axis represents the first CIA principal component (explaining 74.7%, 74.5%, and 71.1% of the variance, respectively), and the vertical axis represents the second principal component (explaining 11.9%, 15%, and 18% of the variance, respectively). Samples from the three groups are color-coded, circles represent metabolite clusters, triangles represent behavioral phenotype clusters, and the distance between points reflects association strength. The RV coefficient quantified the overall association between the metabolome and phenotypes. *p < 0.05, **p < 0.01, and ***p < 0.001.

To investigate alterations in energy metabolism underlying CSDS-induced depression-like behaviors and the regulatory effects of FMT, we performed targeted metabolomic analysis of serum, prefrontal cortex (PFC), and hippocampus (HIP) (Figure 7A, Table S12). Compared to the CON group, the CSDS group exhibited a significant decrease in D(+)-glucose levels in serum, PFC, and HIP. Concurrently, several glycolytic intermediates (glucose‑6‑phosphate, fructose‑6‑phosphate, fructose‑1,6‑bisphosphate, glyceraldehyde‑3‑phosphate) were significantly elevated in these compartments. In the PFC of CSDS mice, key TCA cycle intermediates (citrate, α‑ketoglutarate, fumarate) were also significantly increased. Levels of the energy carriers ATP and NAD were significantly reduced in the brain regions of CSDS mice. Lactate levels were specifically elevated in the CSDS group, with the most pronounced increase observed in the PFC. In the CSDS+FMT group, these metabolic alterations were markedly attenuated. D(+)‑Glucose levels recovered toward CON levels, the accumulation of glycolytic intermediates was reduced, and the elevated levels of TCA cycle intermediates in the PFC were normalized. The levels of ATP and NAD in brain regions showed a recovery trend. Furthermore, the abnormally elevated lactate levels in serum and brain were significantly reduced following FMT intervention. For several metabolites (e.g., certain TCA cycle intermediates and glucose‑6‑phosphate), the significant difference from the CON group was abolished after FMT. In addition, the CSDS group displayed significant alterations in urea‑cycle‑related metabolites (e.g., arginine, ornithine, and L‑citrulline). L‑Glutamate, a downstream metabolite of arginine, was markedly elevated in serum and brain regions of CSDS mice, consistent with our observations in the human cohort. FMT intervention normalized the levels of these urea cycle metabolites and glutamate toward those of the CON group.

Correlation analysis further revealed coordinated alteration patterns between energy metabolism disturbances across different tissues and behavioral phenotypes in mice (Figure 7B). Specifically, D(+)-glucose, ATP, and NAD levels in serum, PFC, and HIP showed positive correlations with multiple behavioral indicators (e.g., OFT, SPT, and NOR), suggesting that deficiencies in these key energy metabolites may directly contribute to behavioral impairments. In contrast, lactate and L-glutamate were negatively correlated with NOR performance, indicating that their abnormal elevation may underlie behavioral deficits such as social avoidance and reduced exploratory behavior. Among urea cycle-related metabolites, serum arginine levels correlated positively with behavioral performance; its reduction may exacerbate behavioral abnormalities by compromising immunomodulatory and neuroprotective functions. These association patterns suggest that CSDS-induced metabolic disruptions do not occur in isolation, but rather act in concert, exemplified by the synergistic negative effects of lactate and L-glutamate, together with the combined deficiency in ATP and arginine, to collectively drive the emergence of abnormal behavioral phenotypes.

CIA was further employed to jointly project data on metabolites and behavioral phenotypes from the three mouse groups into a two-dimensional space, with the RV coefficient used to evaluate the strength of association between the two datasets. The results further validated the impact of energy metabolism dysregulation on behavioral phenotypes and the restorative effect of FMT (Figure 7C). In terms of sample distribution, the CON group exhibited tightly clustered patterns in serum, PFC, and HIP, indicating a stable metabolism–behavior relationship in healthy mice. In contrast, the CSDS group samples significantly deviated from the CON group and showed the most dispersed distribution in the PFC, reflecting that CSDS not only disrupted the intrinsic link between metabolism and behavior but also increased metabolic heterogeneity in this brain region. By comparison, the CSDS-FMT group samples shifted noticeably toward the CON group, visually demonstrating that FMT restored the normal association pattern between metabolism and behavior by correcting aberrant levels of key metabolites such as lactate, ATP, and L-glutamate. Regarding the RV coefficients across different compartments, both serum (RV = 0.402) and PFC (RV = 0.408) showed higher values than HIP (RV = 0.300), suggesting that serum metabolites may serve as peripheral markers of metabolism–behavior associations, with their changes effectively reflecting the systemic metabolic impact on behavior. As a key region for emotional and cognitive regulation, the PFC exhibited a tighter metabolism–behavior coupling, further supporting the existence of a “peripheral metabolic disruption–brain metabolic imbalance–abnormal behavioral phenotype” regulatory axis.

**Figure 8.**
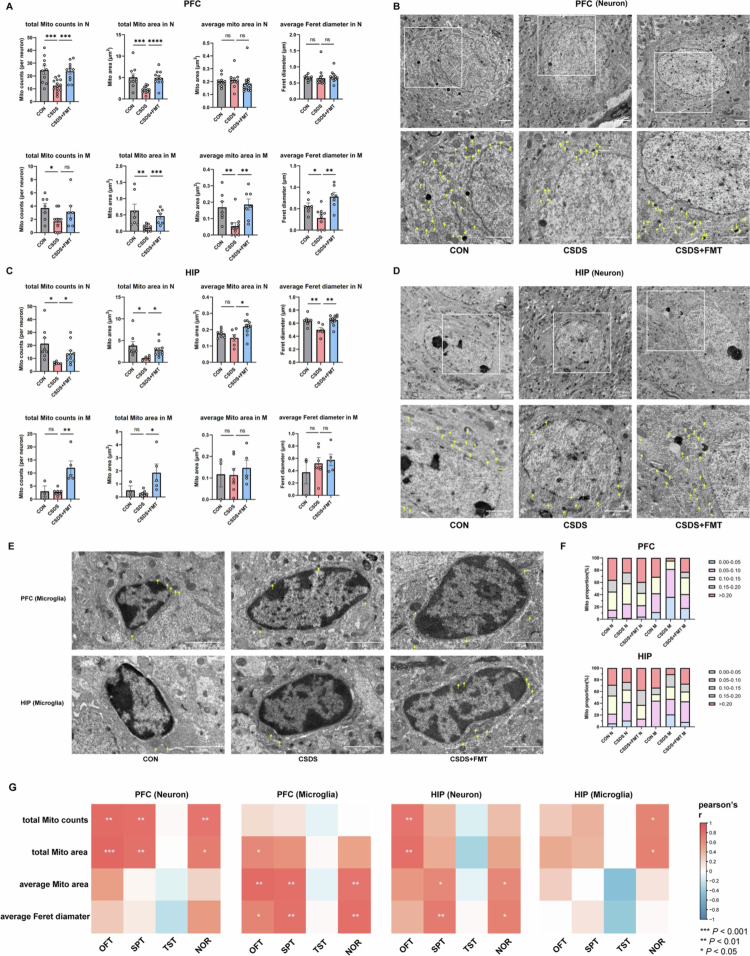
Effects of CSDS and FMT on mitochondria in neurons and microglia of mice and their behavior associations. (A and C) Quantitative analysis of mitochondria in neurons (N) and microglia (M) in the PFC and HIP of mice across the three groups under transmission electron microscopy (TEM), including total number, total area, average area, and average Feret diameter. (B, D, and E) Representative TEM images showing mitochondrial morphology in neurons and microglia in the PFC and HIP across the three groups. The white scale bar in the lower right indicated 2 μm; yellow arrows pointed to mitochondria. (F) Stacked bar charts illustrating the proportional composition of mitochondria classified by size in neurons and microglia in the PFC and HIP across the three groups under TEM. The inset indicated the mitochondrial area range (unit: μm²) represented by each color. (G) Correlation analysis between mitochondrial parameters in neurons and microglia in the PFC and HIP under TEM and behavioral phenotypes across the three groups.

Metabolomic data from serum, PFC, and HIP consistently indicated that chronic stress drives a systemic and local cerebral shift toward anaerobic glycolysis, likely mediated by mitochondrial dysfunction. To investigate this, we used transmission electron microscopy (TEM) to examine the effects of CSDS and FMT intervention on mitochondrial morphology and ultrastructure in neurons and microglia of the PFC and HIP. As shown in Figure 8A and C, the total number and total area of mitochondria in neurons were significantly greater than those in microglia in both brain regions, while the average area and mean maximum Feret diameter of mitochondria remained comparable between the two cell types. Compared with the CON group, CSDS significantly reduced the total number and total area of mitochondria in both neurons and microglia in the PFC. In microglia, the average mitochondrial area and mean maximum Feret diameter were also reduced, whereas these parameters remained unchanged in neurons. FMT treatment effectively reversed these abnormalities, restoring mitochondrial number and size to near-normal levels (Figure 8A). Similarly, in the HIP, CSDS led to significant reductions in the total number, total area, and mean maximum Feret diameter of neuronal mitochondria, and FMT intervention again exerted a significant restorative effect. In contrast, mitochondrial alterations in HIP microglia were less pronounced (Figure 8C). Representative TEM images visually illustrated these morphological changes (Figure 8B, D, and E). Mitochondria in both neurons and microglia of the CON group exhibited intact structures with clearly visible cristae. In the CSDS group, a subset of neuronal mitochondria appeared shrunken, with disrupted or vacuolated cristae, particularly evident in the HIP. Mitochondria in microglia showed shortening and cristae blurring. In the CSDS-FMT group, mitochondrial morphology and structural integrity were substantially restored in both cell types. Further sub-analysis of mitochondria by size revealed that the CSDS group showed an increased proportion of smaller mitochondria (<0.10 μm²) and a decreased proportion of larger mitochondria (>0.20 μm²) (Figure 8F). The mitochondrial size distribution in the CSDS-FMT group closely resembled that of the CON group. Pearson correlation analysis demonstrated that total mitochondrial number, total area, average area, and mean maximum Feret diameter were all positively correlated with behavioral performance (Figure 8G). Specifically, in the PFC, both the total number and total area of neuronal mitochondria correlated positively with OFT, SPT, and NOR performance. In PFC microglia, mitochondrial average area and mean maximum Feret diameter were also positively correlated with these behavioral measures. In the HIP, the total number and total area of neuronal mitochondria showed positive correlations with OFT performance, while average area and mean maximum Feret diameter correlated with SPT and NOR. For microglial mitochondria in the HIP, only total number and total area were positively correlated with NOR performance, with no significant associations observed with OFT or SPT. These findings indicate that the structural integrity of central mitochondria regulates behavioral function in a brain region- and cell type-specific manner. Both the behavioral deficits and mitochondrial damage induced by CSDS were effectively rescued by FMT.

## Discussion

The pathophysiological alterations and pathogenic mechanisms of MDD have consistently been a focal and challenging area of research. The role of the gut microbiota in MDD via the microbiota–gut–brain axis (MGBA) has garnered increasing attention. Notably, dysregulation of energy metabolism is widely recognized as a core pathological feature of MDD, manifesting as disturbances in key pathways such as glycolysis, the TCA cycle, and amino acid metabolism.^30^ These alterations may impact central nervous system function through microbiota-mediated metabolic regulation. In this study, by integrating targeted metabolomics with shotgun metagenomic sequencing, we first revealed in a human cohort a regulatory axis through which the gut microbiota influences depressive symptoms via energy metabolites. Furthermore, utilizing a CSDS mouse model, we validated the role of autologous FMT in reversing metabolic imbalances and improving behavioral phenotypes.

We observed significantly reduced glucose levels in 100 patients with MDD, which could potentially be attributed to appetite loss associated with low mood. During mitochondrial dysfunction, impaired glucose catabolism disrupts the TCA cycle and promotes anaerobic glycolysis, ultimately leading to lactate accumulation. This suggests that patients with MDD are often in a state of insufficient ATP, resulting in neuronal energy deficiency and exacerbation of depressive symptoms. Moreover, high lactate concentrations in brain tissue may induce metabolic acidosis, a pathological process that can cause apoptotic pathways and contribute to irreversible neuronal damage.^31^ Within the TCA cycle, we observed a significant increase in isocitric acid levels. Although there is currently insufficient evidence to support the finding that isocitrate levels are generally reduced during depression, Chen et al. reported a negative correlation between serum isocitric acid levels and Childhood Trauma Questionnaire scores in adolescent patients with MDD.^32^ Furthermore, we observed high levels of α-ketoglutaric acid and L-glutamic acid in patients with MDD. α-Ketoglutaric acid, a ketoacid derivative of glutamate deamination, contributes to the formation of the excitatory neurotransmitter glutamate upon amination. Excess glutamate can have neurotoxic effects and promote neuronal injury.^33^ Disruptions in the ornithine cycle can impair ammonia metabolism, leading to hyperammonemia. Ammonia accumulation disrupts mitochondrial energy metabolism and compromises cerebral function, potentially contributing to fatigue, cognitive decline, and other depression-like symptoms.^34^ Arginine, an important metabolite in the ornithine cycle, is significantly high during depression, which is consistent with previous research results^35^ and has been proven to be involved in the pathogenesis of neurodegenerative diseases. Ornithine serves as a precursor for neuroprotective polyamines (e.g., spermine and spermidine), and its deficiency may increase the risk of depression.^36^ Clinical trials have demonstrated that L-ornithine supplementation significantly improves fatigue and anger scores.^37^ Moreover, patients with MDD exhibited significantly reduced levels of adenine, AMP, cyclic AMP, guanosine, and uracil, indicating impaired purine and pyrimidine metabolism. The decrease in adenine, an AMP precursor, may have resulted from suppressed purine synthesis or accelerated degradation. Reduced levels of AMP, a critical intermediate in ATP production, further reflect cellular energy dysregulation.^38^ Diminished cAMP, a secondary messenger, attenuates protein kinase A activity, thereby impairing brain-derived neurotrophic factor (BDNF) signaling and reducing neuroplasticity and synaptogenesis.^39^ cAMP and guanosine modulate glutamatergic excitability,^40^ potentially exacerbating the cognitive deficits in patients with depression. Notably, these purine and pyrimidine metabolites showed significant negative correlations with depressive symptom severity, suggesting that patients with MDD may exhibit enhanced compensatory nucleotide metabolism in response to an aberrant energy supply.

The gut microbiota constitutes a complex ecological community characterized by competitive and symbiotic interactions, and is pivotal in regulating mood and cognitive function during depression. According to PCoA results, significant differences were observed in the overall structure of the gut microbiota between patients with MDD and healthy individuals. At the phylum level, significantly increased relative abundances of Proteobacteria, Candidatus Saccharibacteria, and Verrucomicrobia were observed in patients with MDD, consistent with previous reports. Proteobacteria have been frequently identified as a dominant phylum in MDD,^27^^,^^41^ and its elevated abundance may exacerbate depressive symptoms through metabolic dysregulation. Candidatus Saccharibacteria, which is highly concentrated in the caries-associated oral microbiota,^42^ suggests potential oral gut microbial dysfunction in patients with MDD. This is consistent with the evidence of a higher oral disease burden in patients with depression^43^ and possible translocation of oral bacteria to the gut. The high concentration of Verrucomicrobia in patients seems to be species-specific.^44^ Preclinical studies have demonstrated that puerarin alleviates depression-like behaviors in rats with chronic unpredictable mild stress and reduces Verrucomicrobia abundance. Notably, Verrucomicrobia abundance correlates with environmental stressors such as salinity and heavy metal exposure,^45^ implicating its potential role in modulating depression through host-environment interactions, possibly through immune or metabolic mechanisms. Host–microbiota interactions involve multiple critical mediators, with mitochondria emerging as a crucial interface linking gut dysbiosis to depression.^46^ The gut microbiota can disrupt host energy homeostasis through two principal mechanisms: directly encoding energy metabolism-related enzymes homologous to the host, and modulation of amino acid metabolism.^47^^,^^48^ Our findings demonstrated that disordered microbial communities in patients with MDD significantly altered multiple energy metabolic mechanisms, such as succinate/lactate consumption and proline/arginine/lysine degradation. Notably, *Escherichia_coli* and *Klebsiella_pneumoniae*, which were significantly concentrated in the MDD group, highly expressed enzymes in the TCA cycle, thereby regulating the homeostatic balance of host energy metabolism intermediates. In an antibiotic-induced depression mouse model, the *Klebsiella* abundance value significantly increases, whereas the levels of 5-HT and BDNF in the hippocampus and prefrontal cortex significantly decrease.^49^ Combined with the results of energy metabolism in this study, it is suggested that the intestinal microbiota may competitively consume the host’s metabolic substrates or secrete specific metabolites, interfering with the homeostasis of intermediate products in the TCA cycle (e.g., α-ketoglutaric acid and succinic acid), and leading to abnormal synthesis of neurotransmitters.^50^ Furthermore, our study elucidated the critical mediating role of energy metabolites in the gut microbiota-cognitive dysfunction axis. Among them crucial metabolites, such as isocitric acid, L-citrulline, lactate, and AMP, were found to mediate the regulatory effects of specific bacterial taxa (e.g., *Dorea formicigenerans* and *Fusicatenibacter saccharivorans*) on depressive symptoms and cognitive impairment by modulating neurotransmitter metabolism, oxidative stress, and energy homeostasis. Although mechanistic studies investigating the precise regulatory mechanisms linking these specific gut microbes to energy metabolites and cognitive function remain insufficient, emerging evidence from associated studies and animal experiments have established their potential relationships.^51^^,^^52^ Notably, *Fusicatenibacter saccharivorans*, a short-chain fatty acid (SCFA)-producing bacterium, is significantly depleted in patients with MDD^53^ and positively correlated with better cognitive performance.^54^ Furthermore, SCFAs (e.g., acetic acid, propionic acid, and butyric acid) are the main energy source for colonic epithelial cells, generating ATP through β-oxidation and the TCA to provide energy for the entire body.^55^ Moreover, as histone deacetylase inhibitors, SCFAs epigenetically regulate the expression of genes involved in lipid synthesis and energy-sensing mechanisms (e.g., AMPK), thereby suppressing lipogenesis while promoting energy expenditure.^56^ These findings strongly support the diverse regulatory framework of the gut microbiota-metabolite-brain axis, particularly in terms of energy metabolism reprogramming and neuroplasticity modulation, forming a mechanistic loop.^57^^,^^58^

To establish the causal relationship of the “gut microbiota–energy metabolite–depressive phenotype” axis, this study employed a CSDS mouse model for intervention with autologous healthy FMT. The results demonstrated that FMT effectively reshaped the gut microbial structure, significantly improved depression-like behaviors, and reversed the anaerobic glycolytic shift in both serum and brain regions. More importantly, FMT restored mitochondrial morphology and structural integrity in the prefrontal cortex and hippocampus, re-establishing the normal association between metabolic markers and behavioral phenotypes. Previous animal studies have similarly shown that transplanting gut microbiota from MDD patients into germ-free mice can induce depressive behaviors accompanied by hepatic metabolic abnormalities,^59^ whereas transplantation of a “healthy microbiota” can alleviate such metabolic disturbances and behavioral deficits.^60^^,^^61^ By focusing on central energy metabolism (e.g., mitochondrial repair), our work extends these findings and emphasizes that FMT not only modulates the intestinal environment but also directly improves cerebral metabolic homeostasis. The therapeutic efficacy of FMT stems from its multi-target actions, potentially correcting energy metabolism imbalances through multiple pathways. For instance, healthy microbiota transplantation can increase the production of SCFAs, activate the GPR41 receptor, and promote glucagon-like peptide-1 (GLP-1) secretion, thereby improving glucose metabolism and mitochondrial biogenesis.^62^ This aligns with the normalization of metabolic indicators observed in our study, such as the decrease in lactate levels reflecting suppressed glycolysis. Furthermore, a healthy microbiota may indirectly ameliorate energy metabolism by attenuating neuroinflammation and oxidative stress.^63^ Neuroimaging studies have revealed that cognitive impairment in MDD patients is associated with altered hippocampal function and is influenced by the gut microbiota.^64^ Our observation that FMT reduces lactate accumulation and restores hippocampal mitochondrial structure supports the hypothesis that the microbiota enhances neuroplasticity by improving energy supply. Although FMT represents a promising intervention strategy for MDD, and other microbiota-targeted interventions (e.g., probiotics) have shown some efficacy in MDD,^65^ their specificity requires further optimization. The autologous FMT strategy adopted in this study preserves host specificity, avoids potential rejection risks associated with allogeneic transplantation, and achieves more comprehensive and stable microbial ecological reconstruction.

While this study provides strong evidence for the role of the microbiota-metabolism axis in MDD, certain limitations should be acknowledged. First, the relatively limited sample size (100 patients) may affect statistical power and generalizability of the findings. Although our diagnostic model demonstrated high accuracy in an internal test set, its exceptional performance requires validation in an independent, external cohort to confirm robustness and mitigate potential overfitting. A systematic review noted that inconsistencies in MDD microbiota research are often due to sample heterogeneity.^66^ Therefore, future multi-center, large-sample studies are needed both to replicate the microbiota-metabolite associations and to externally validate the diagnostic model. Second, some mechanistic details remain incompletely elucidated, such as how specific metabolites (e.g., lactate) precisely mediate gut–brain signaling. Furthermore, this study did not deeply explore the interaction between metabolism and the immune system (e.g., changes in cytokine levels), whereas existing literature indicates that inflammation is a key component of the MGBA. Subsequent research could integrate multi-dimensional data, such as proteomics, to comprehensively dissect the interactive networks among metabolism, immunity, and neural circuits.

## Conclusion

Through integrated multi-omics analysis and animal validation, this study systematically reveals the central role of the gut microbiota in MDD via the regulation of host energy metabolism, establishing a “microbiota–metabolite–depressive phenotype” regulatory axis. Key findings include: disturbances in energy metabolism products (e.g., lactate, glutamate) in MDD patients correlate with symptom severity; specific gut bacteria influence behavior through mediating effects; and early autologous FMT can reverse central metabolic imbalance and mitochondrial damage. These results not only deepen the understanding of the MGBA mechanisms but also offer a new clinical strategy—targeting the restoration of the microbiota–metabolism axis, particularly by correcting central energy metabolism dysregulation through early autologous FMT, which may serve as a potential novel approach to overcoming current therapeutic limitations.

## Supplementary Material

Supplementary material figures.

Supplementary_information clean.docx Supplementary_information clean.docx

Supplemental material Supplemental table S1_s12.xlsx

## Data Availability

The metagenomic sequencing data reported in this paper can be found in the Genome Sequence Archive database (CRA019246).
