# Supplementary material for: Gut microbiota reshapes host energy metabolism to modulate depressive behaviors
Source: Gut Microbes. 2026 Apr 23;18(1):2662556. doi: 10.1080/19490976.2026.2662556 (PMC13108357; doi:10.1080/19490976.2026.2662556)
Supplement: Supplementary material — figures. [file KGMI_A_2662556_SM7448.zip › figure S1.pdf]

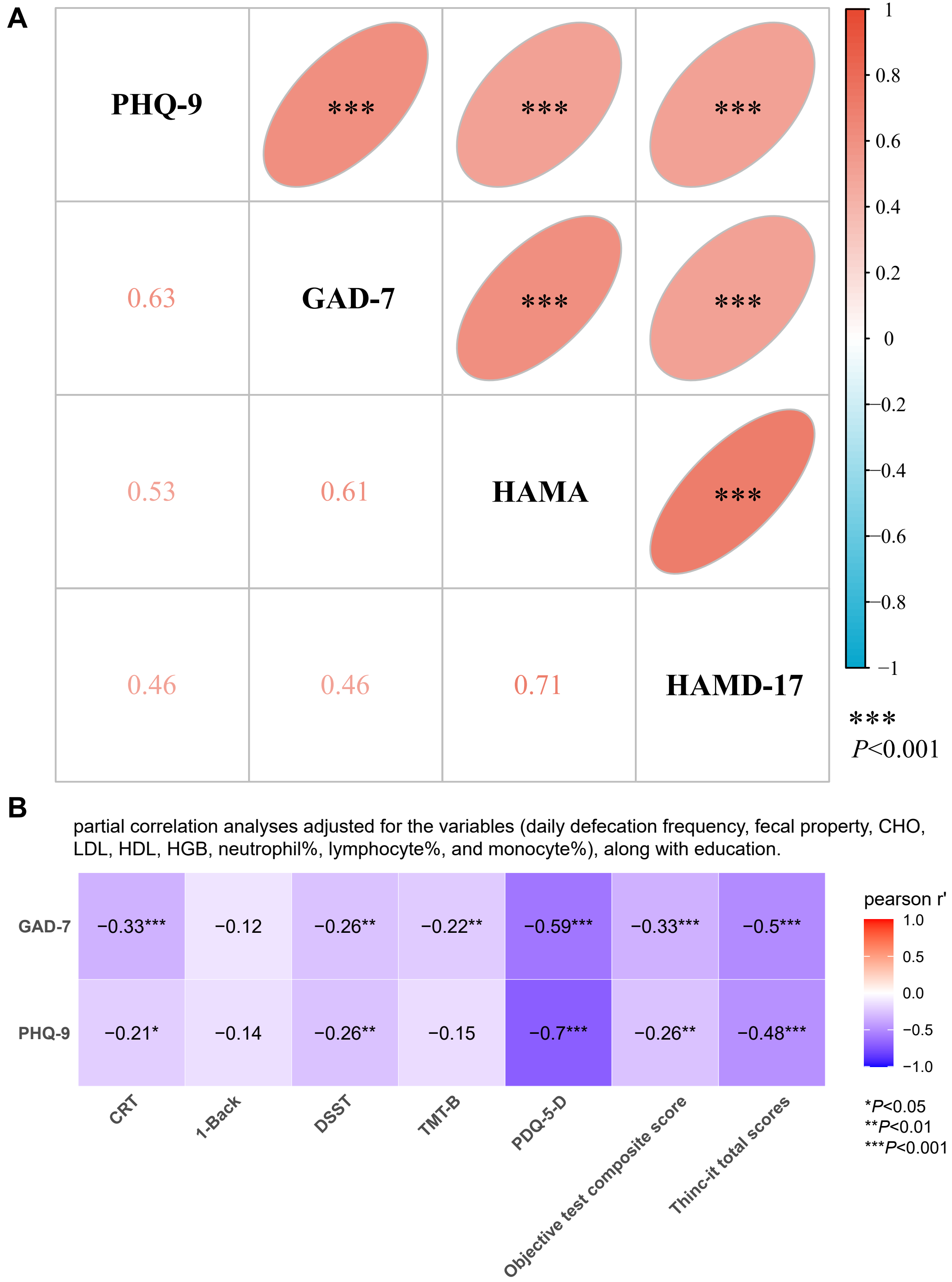

A
PHQ-9
GAD-7
HAMA
HAMD-17
***
0.63
0.53
0.61
0.46
0.46
0.71
1
0.8
0.6
0.4
0.2
0
−0.2
−0.4
−0.6
−0.8
−1
*** P<0.001
B
partial correlation analyses adjusted for the variables (daily defecation frequency, fecal property, CHO, LDL, HDL, HGB, neutrophil%, lymphocyte%, and monocyte%), along with education.
GAD-7
PHQ-9
−0.33***
−0.12
−0.26**
−0.22**
−0.59***
−0.33***
−0.5***
−0.21*
−0.14
−0.26**
−0.15
−0.7***
−0.26**
−0.48***
CRT
1-Back
DSST
TMT-B
PDQ-5-D
Objective test composite score
Thinc-it total scores
pearson r'
1.0
0.5
0.0
−0.5
−1.0
*P<0.05
**P<0.01
***P<0.001
