# Supplementary material for: Gut microbiota reshapes host energy metabolism to modulate depressive behaviors
Source: Gut Microbes. 2026 Apr 23;18(1):2662556. doi: 10.1080/19490976.2026.2662556 (PMC13108357; doi:10.1080/19490976.2026.2662556)
Supplement: Supplementary material — figures. [file KGMI_A_2662556_SM7448.zip › figure S10.pdf]

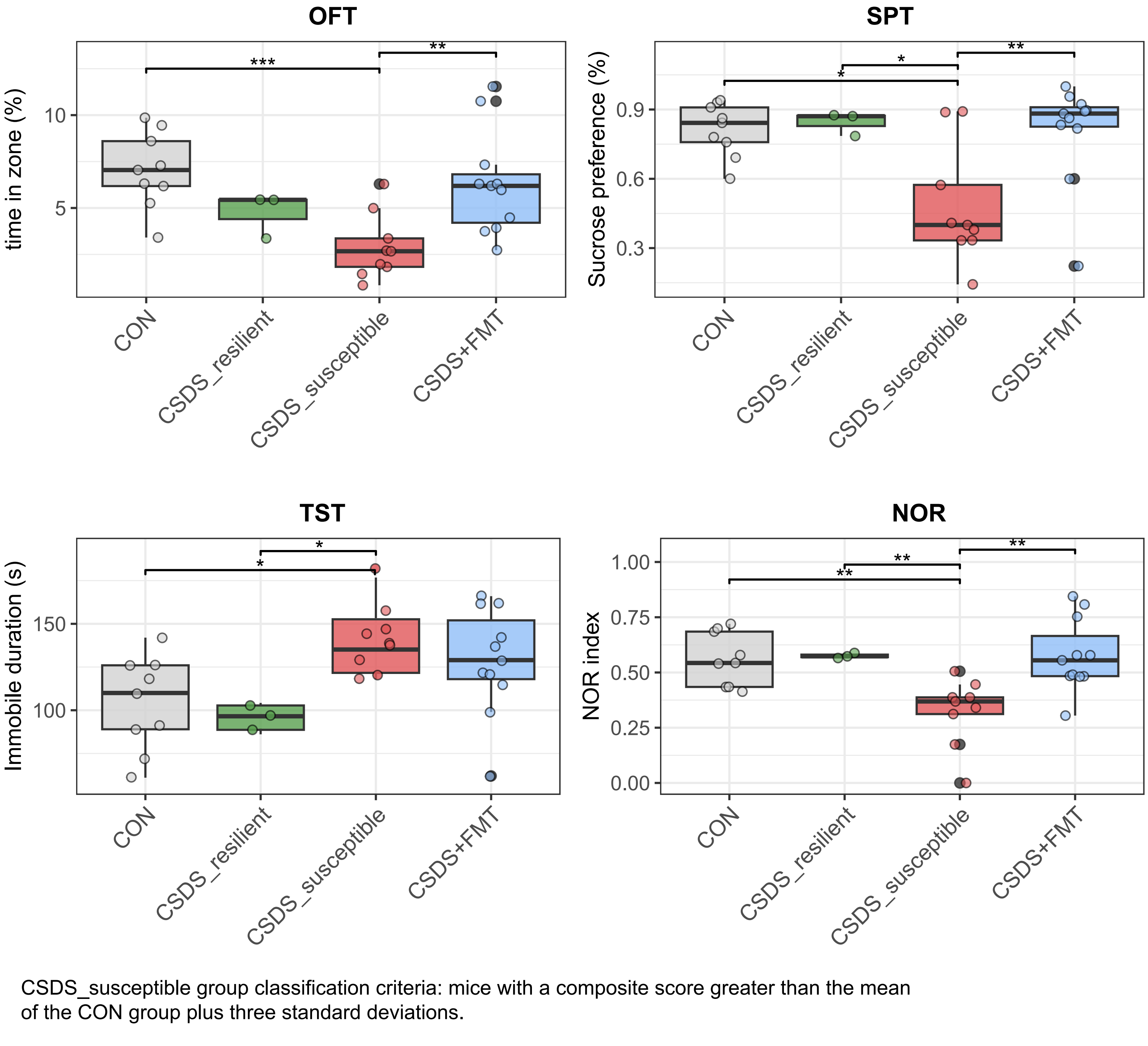

CSDS_susceptible group classification criteria: mice with a composite score greater than the mean of the CON group plus three standard deviations.
