# Supplementary material for: Gut microbiota reshapes host energy metabolism to modulate depressive behaviors
Source: Gut Microbes. 2026 Apr 23;18(1):2662556. doi: 10.1080/19490976.2026.2662556 (PMC13108357; doi:10.1080/19490976.2026.2662556)
Supplement: Supplementary material — figures. [file KGMI_A_2662556_SM7448.zip › figure S11.pdf]

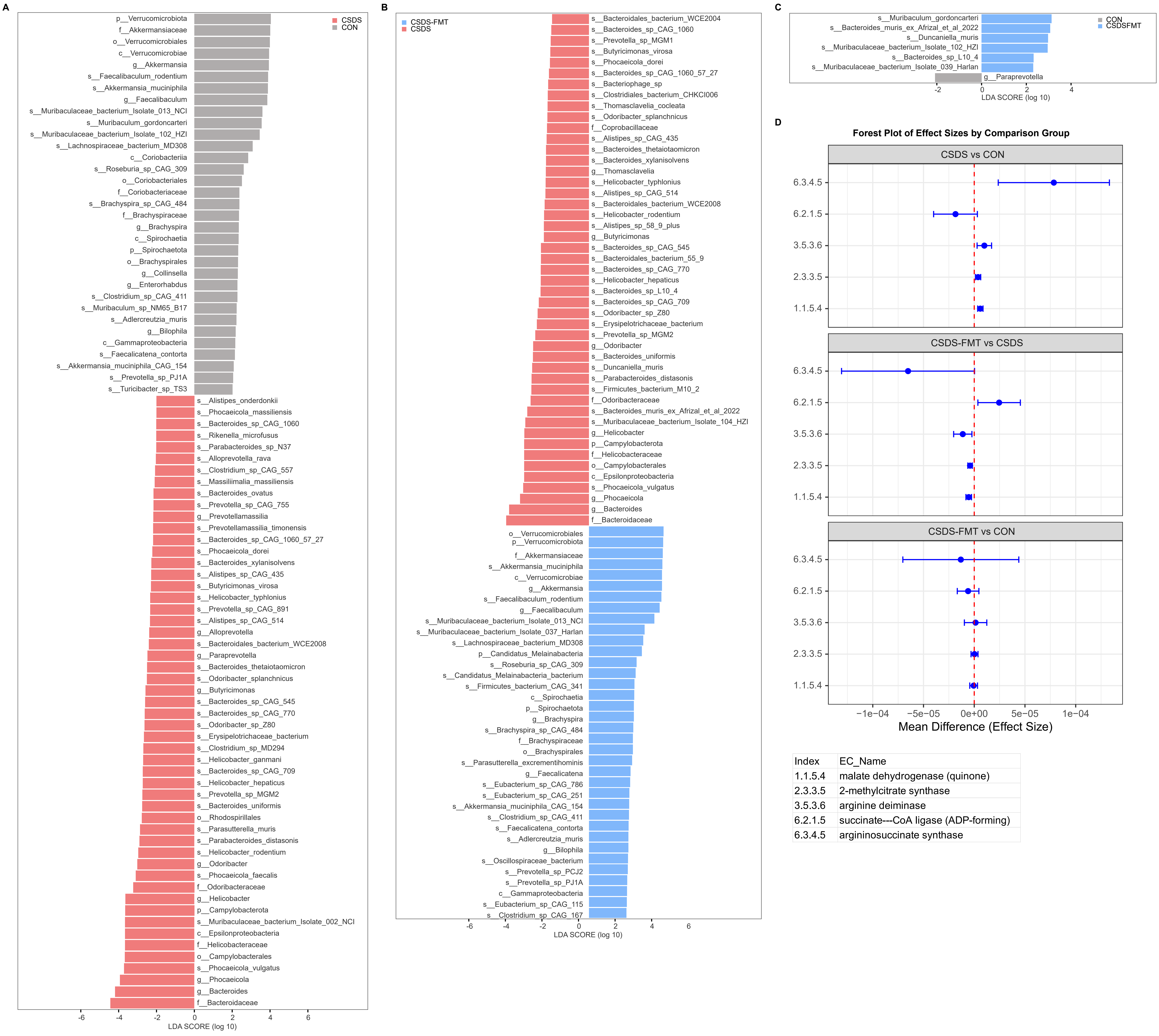

Index	EC_Name
1.1.5.4	malate dehydrogenase (quinone)
2.3.3.5	2-methylcitrate synthase
3.5.3.6	arginine deiminase
6.2.1.5	succinate---CoA ligase (ADP-forming)
6.3.4.5	argininosuccinate synthase
