# Supplementary figures and images for: Gut microbiota reshapes host energy metabolism to modulate depressive behaviors
Source: Gut Microbes. 2026 Apr 23;18(1):2662556. doi: 10.1080/19490976.2026.2662556 (PMC13108357; doi:10.1080/19490976.2026.2662556)

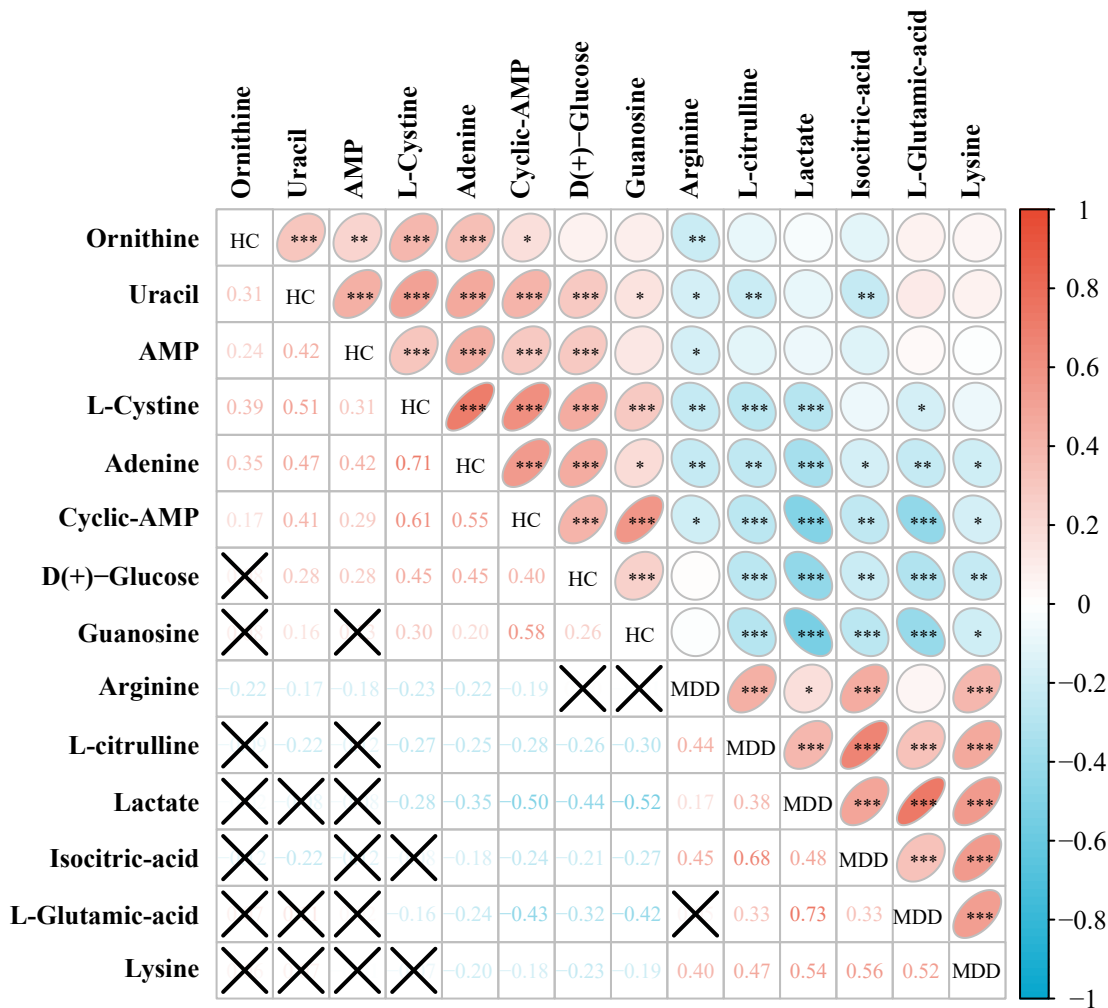

Supplement: Supplementary material — figures. [file KGMI_A_2662556_SM7448.zip › figure S2.pdf]

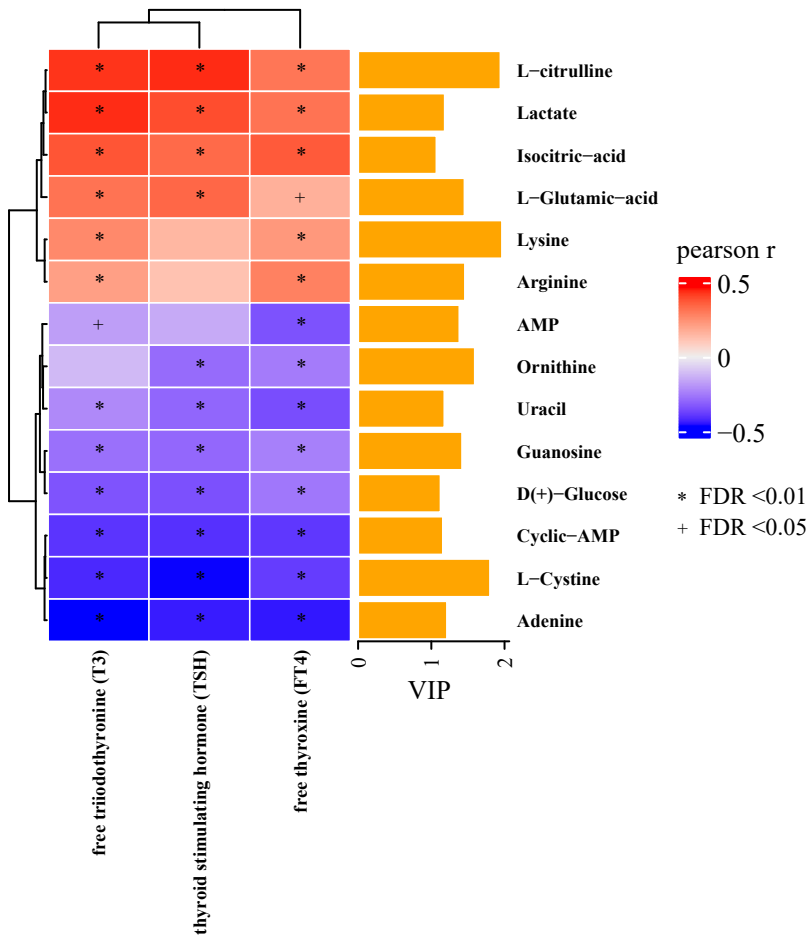

Supplement: Supplementary material — figures. [file KGMI_A_2662556_SM7448.zip › figure S3.pdf]

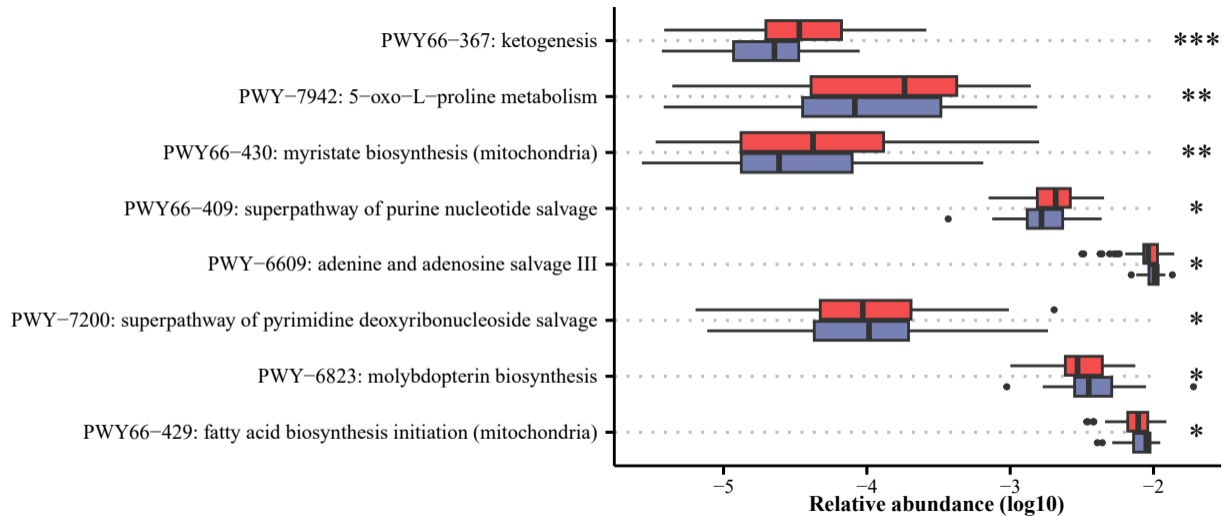

Supplement: Supplementary material — figures. [file KGMI_A_2662556_SM7448.zip › figure S4.pdf]

# PWY66-429: fatty acid biosynthesis initiation (mitochondria)

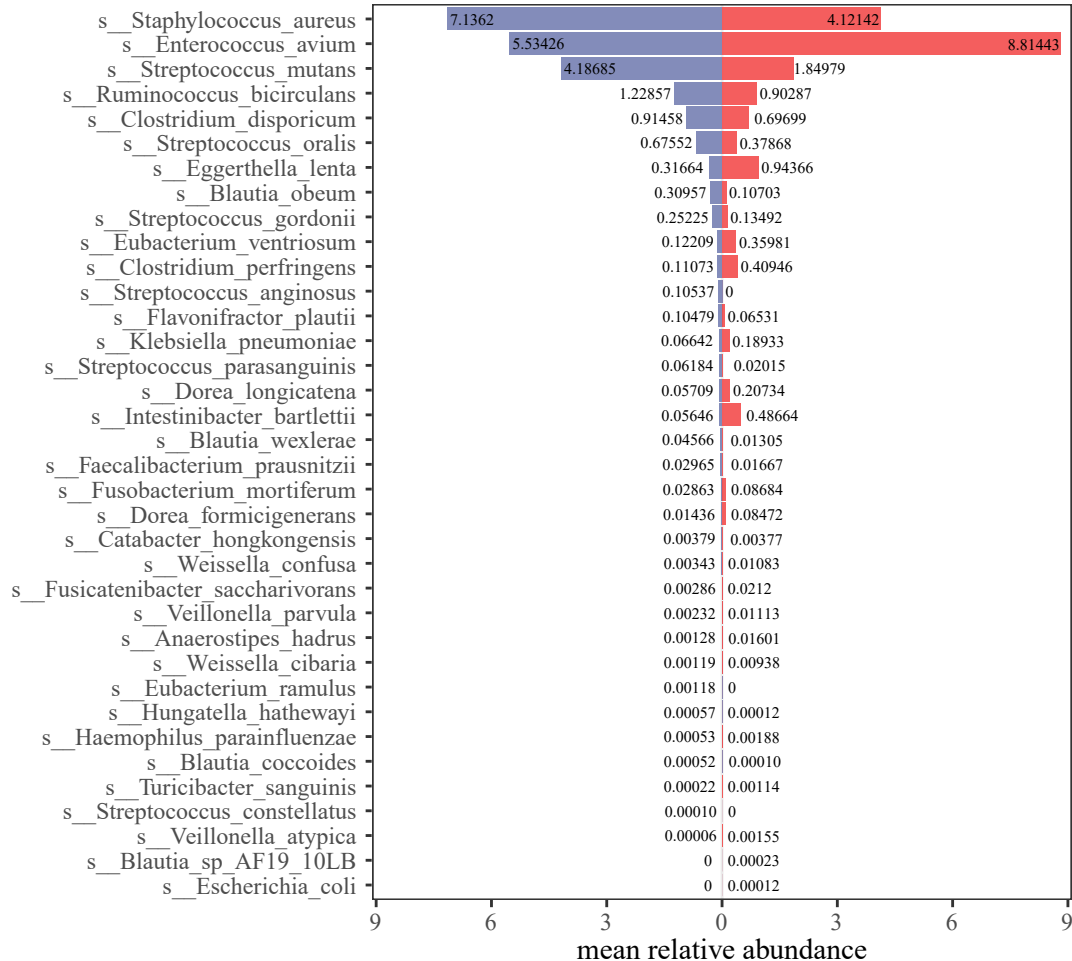

Supplement: Supplementary material — figures. [file KGMI_A_2662556_SM7448.zip › figure S5.pdf]

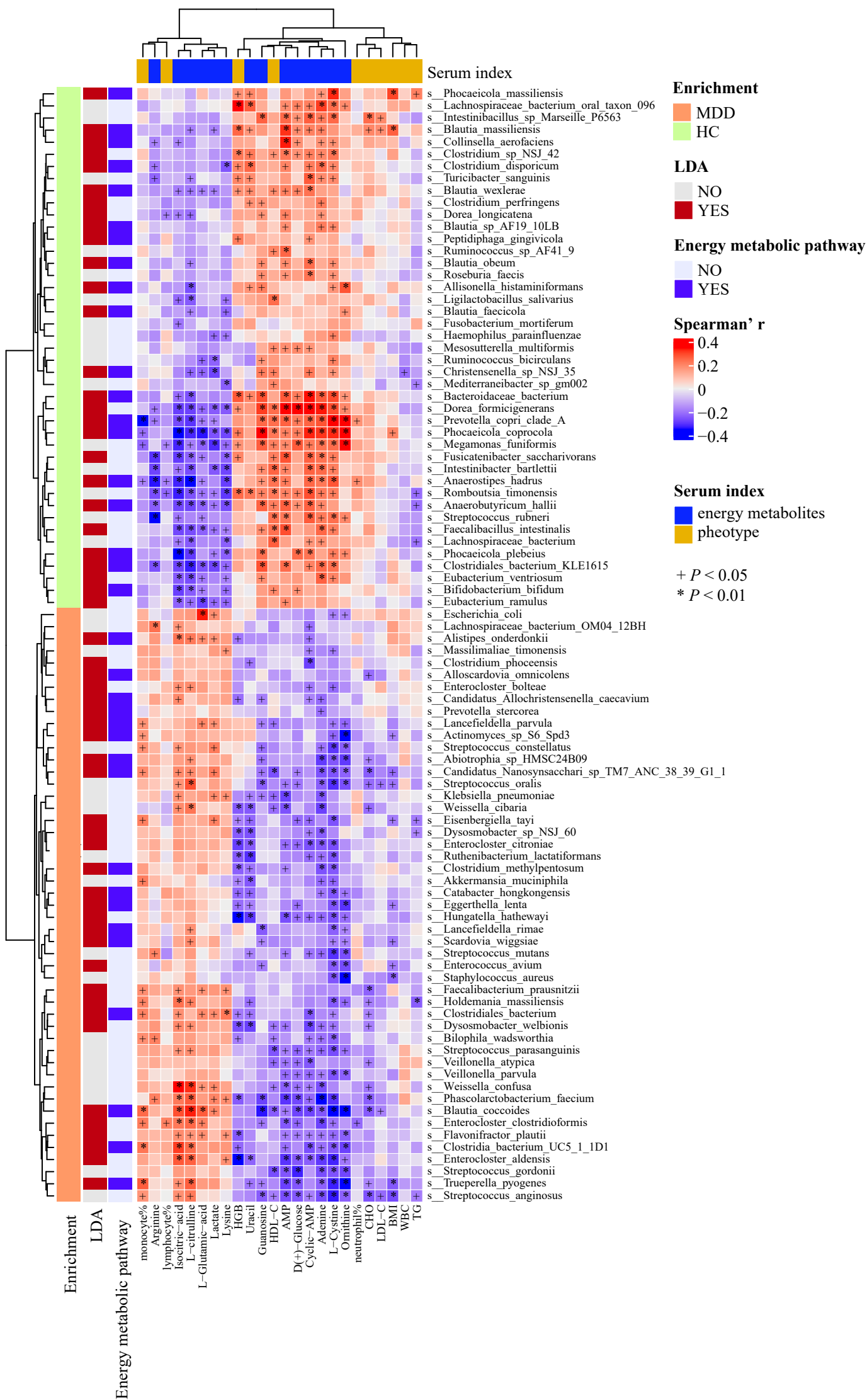

Supplement: Supplementary material — figures. [file KGMI_A_2662556_SM7448.zip › figure S6.pdf]

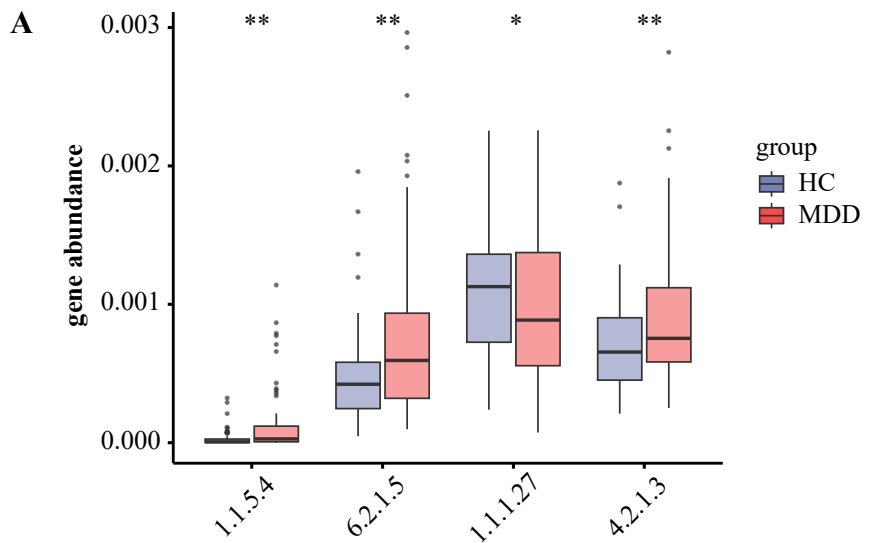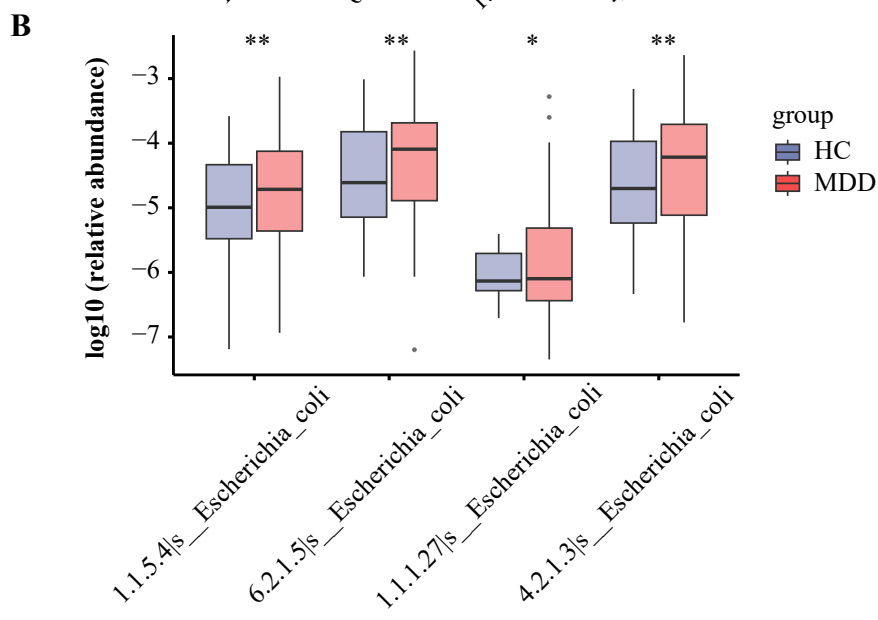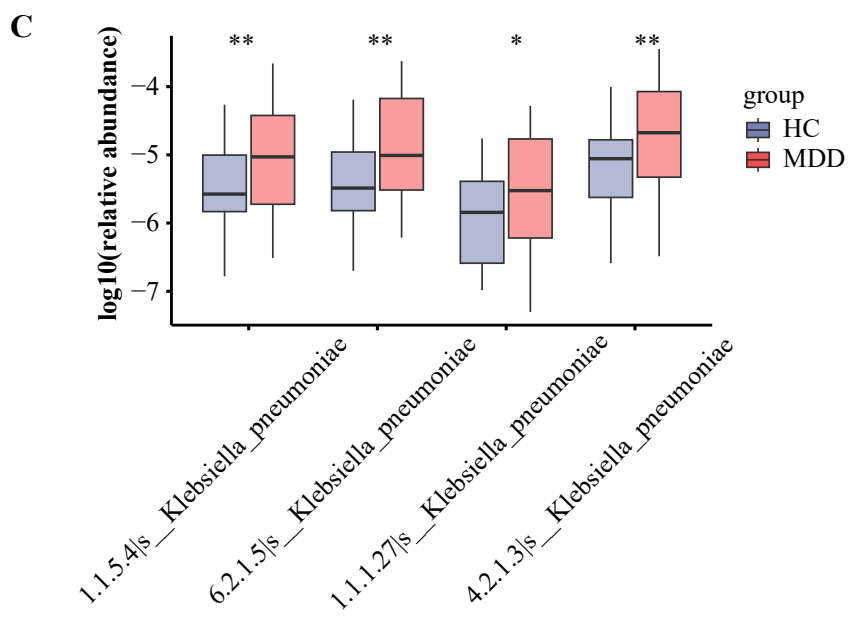

Supplement: Supplementary material — figures. [file KGMI_A_2662556_SM7448.zip › figure S7.pdf]

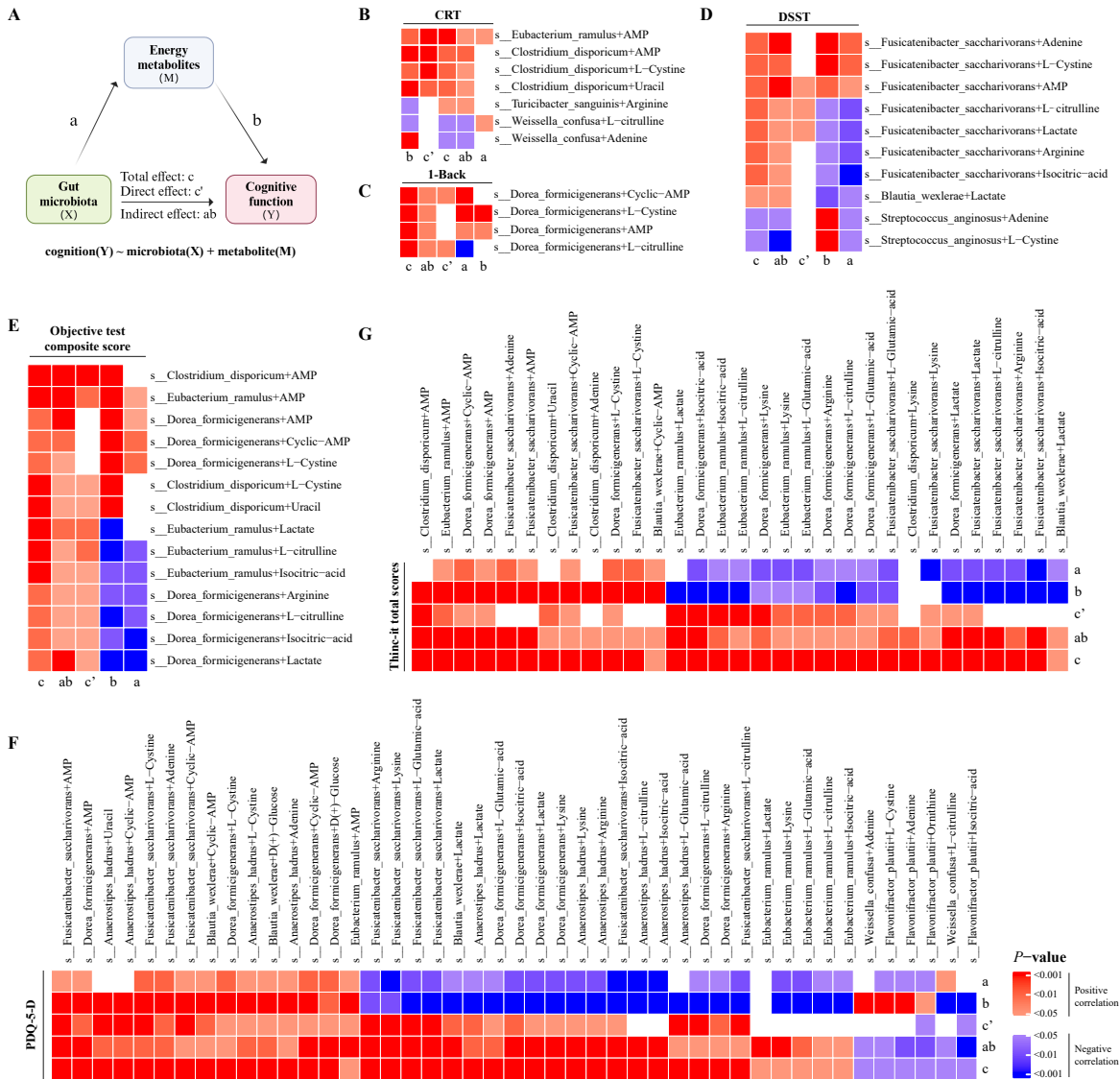

Supplement: Supplementary material — figures. [file KGMI_A_2662556_SM7448.zip › figure S8.pdf]

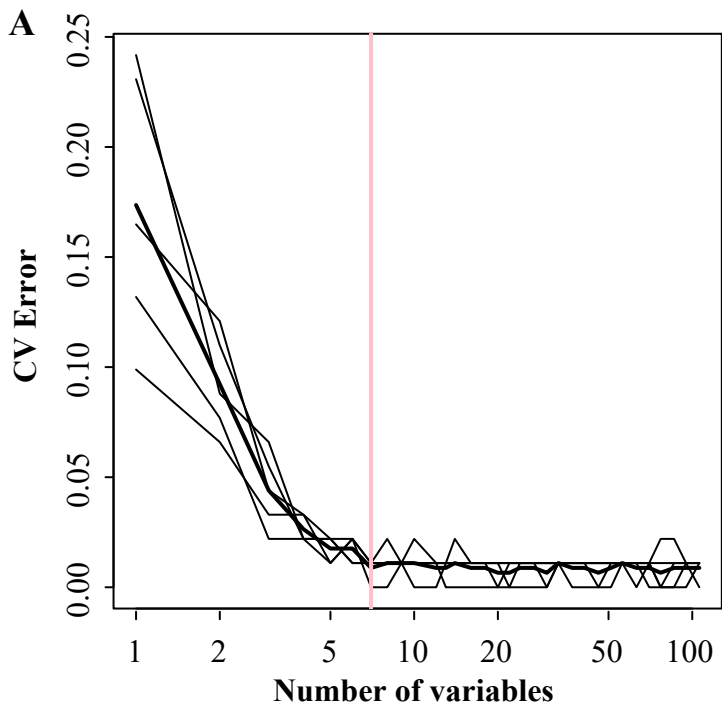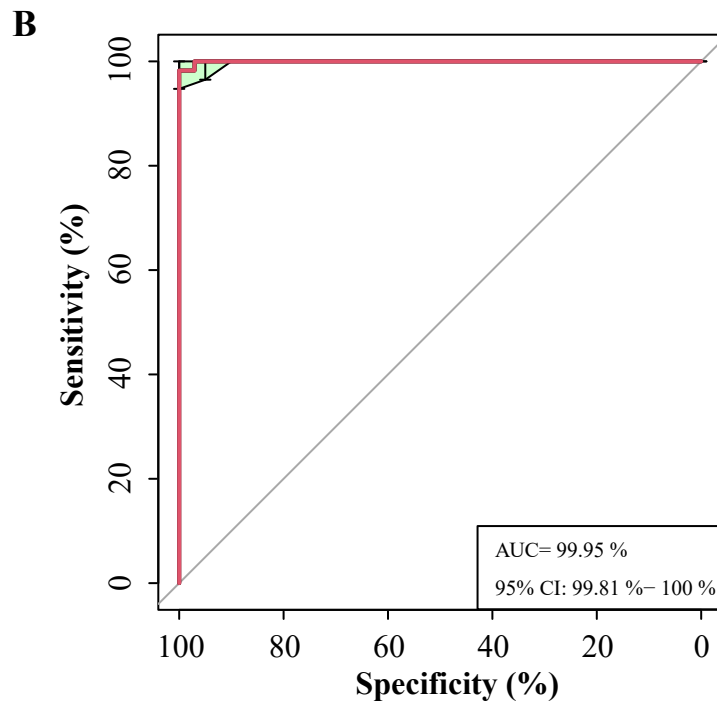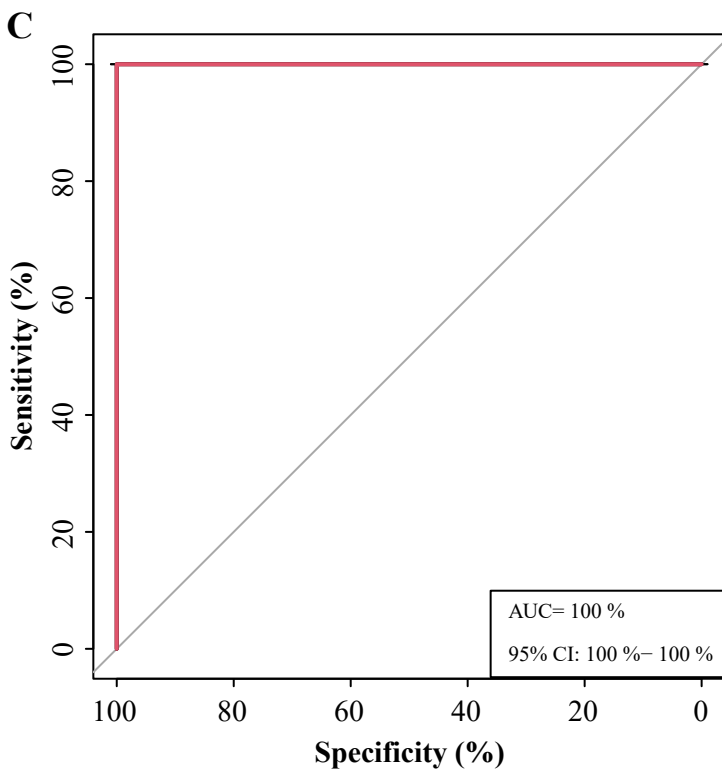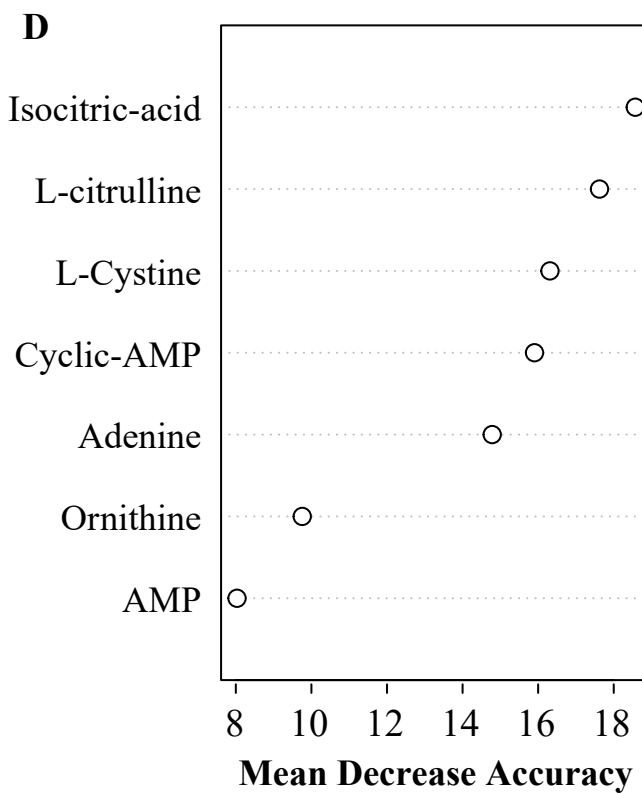

Supplement: Supplementary material — figures. [file KGMI_A_2662556_SM7448.zip › figure S9.pdf]
