# Supplementary material for: Gut microbiota reshapes host energy metabolism to modulate depressive behaviors
Source: Gut Microbes. 2026 Apr 23;18(1):2662556. doi: 10.1080/19490976.2026.2662556 (PMC13108357; doi:10.1080/19490976.2026.2662556)
Supplement: Supplementary_information clean.docx [file KGMI_A_2662556_SM6255.docx]

**Supplementary Material**

**Methodology**

**Characteristics of the course and medication history of patients with depression**

Although the disease duration varied widely (range 1–60 months), a substantial proportion of patients had not received systematic antidepressant treatment despite experiencing depressive symptoms for years. This was attributed to limited awareness of the disease, concerns about medication, or lack of access to psychiatric care. Among the 100 enrolled patients with depression, 47 were treatment‑naïve (had never taken antidepressants), while the remaining 53 had a history of antidepressant use, primarily selective serotonin reuptake inhibitors (SSRIs) or serotonin‑norepinephrine reuptake inhibitors (SNRIs). The mean duration of prior antidepressant treatment was 3.4 months. All patients, however, met the rigorous inclusion criteria regarding recent medication-naive.

**Measurement of energy metabolites**

Ultra-high performance liquid chromatography (UPLC) (Waters ACQUITY H-ClassD) and tandem mass spectrometry techniques were used to detect energy metabolites. The determination was performed on an ACQUITY UPLC BEH Amide column (1.7 µm, 100 mm×2.1 mm i.d.). Mobile phase A was ultrapure water (10mM ammonium acetate, 0.3% ammonia water) and Mobile phase B was 90% acetonitrile/water (v/v). The mobile phase gradient was set at 5:95 (V/V), 30:70 (V/V), 50:50 (V/V), and 5:95 (V/V) for 9.0-11 min A/B, 8 min A/B, 0-1.2 min A/B, and 11.1-15 min A/B, respectively. The mass spectrum conditions mainly included an electrospray ion source temperature 550 ℃, mass spectrum voltage 5500 V in positive ion mode, mass spectrum voltage -4500 V in negative ion mode, and gas curtain gas 35 psi. In Q-Trap 6500+, each ion pair was scanned according to the optimized cluster-de-clustering voltage and collision energy.

A database was developed based on standard products, and qualitative analysis was performed on the data obtained by mass spectrometry. Quantification was performed using multiple response monitoring mode analysis with a triple quadrupole mass spectrometer. After the mass spectrometry data of different samples were obtained, the chromatographic peaks of all target objects were integrated, and a quantitative analysis was performed using standard curves.

The content of each substance in the sample (ng/mL) = c × V₁ / 1000 / V₂

- **c**: Concentration obtained by substituting the sample peak area into the standard curve (ng/mL);
- **V₁**: Total volume of extraction solution (μL);
- **V₂**: Volume of sample aliquoted (mL).

**Experimental animals**

A total of 36 6–7-week-old male C57BL/6 mice were purchased from Beijing Vital River Laboratory Animal Technology Co., Ltd. All mice were housed under specific pathogen-free (SPF) conditions in a controlled environment: temperature 22 ± 1°C, relative humidity 50–60%, and a 12 h light/dark cycle (lights on at 7:00, off at 19:00). Mice were housed 3–4 per cage with free access to food and water. To minimize handling-related stress, after a 3-day acclimation period, each mouse was gently handled for approximately 3 minutes daily. The modeling procedures commenced following one week of acclimatization. All experimental protocols were reviewed and approved by the Biomedical Ethics Committee of Xi'an Jiaotong University Health Science Center and were conducted in accordance with ethical guidelines.

During the acclimation week, fresh fecal samples were collected from all mice and immediately stored at -80°C. On the day of gavage, fecal bacterial suspensions were prepared. Briefly, the pre-frozen fecal samples were thawed and resuspended in sterile saline (0.9% NaCl) at a fixed ratio of 200 mg feces per 2 mL of saline, followed by thorough homogenization until no large particulates remained. The homogenate was then sequentially filtered through sterile 200-, 400-, and 800-mesh sieves to remove particulate debris. The resulting filtrate was collected in a sterile centrifuge tube, vortexed for 5 min, and centrifuged at 600 × g for 5 min to pellet insoluble material. The clarified supernatant was transferred under sterile conditions into an isolation device for subsequent fecal microbiota transplantation into CSDS mice^1, 2^.

**Chronic social defeat stress (CSDS) model and fecal microbiota transplantation (FMT)**

The CSDS paradigm was used to induce depression-like phenotypes by simulating social stress. Eighty male and 25 female CD1 mice (7–8 weeks old) were purchased and housed under SPF conditions, 3–4 per cage, for a 2-week acclimation period. Subsequently, male CD1 mice were singly housed to establish territorial aggression. When the CD1 males reached 12 weeks of age, resident aggressors were screened one week before the start of the formal experiment. C57BL/6 male mice of the same strain and age as the experimental subjects were used as screening intruders. A C57BL/6 intruder was introduced into the cage of a singly housed CD1 mouse, and the CD1's aggressive behavior was observed. The screening criteria were: 1) the latency to the first attack was less than 1 minute, and 2) the CD1 mouse initiated at least three continuous attacks within a 5-minute observation period. CD1 mice meeting these criteria on at least two out of three consecutive screening days were selected as aggressors.

Experimental C57BL/6 mice were randomly assigned to two groups in a 1:2 ratio: a control (CON) group and a CSDS group. Mice were then numbered and weighed. On the first day of modeling, designated aggressors were assigned. Each CSDS subject was physically introduced into the cage of an aggressor for 5–10 minutes of direct confrontation. Subsequently, a perforated transparent divider was inserted to separate the mice, preventing physical contact while maintaining sensory (visual and olfactory) exposure to the aggressor. On the second day, the CSDS subjects were systematically rotated among the aggressors (e.g., the last subject from the previous day was placed with the first aggressor) to prevent habituation and reduced aggression due to familiarity. This rotation protocol continued daily for 14 consecutive days.

Duration the 14-day CSDS procedure, the defeated mice were randomized into two groups in a 1:1 ratio. One group (CSDS+FMT) received the prepared bacterial suspension (approximately 0.2–0.3 mL) via oral gavage 6 hours after each daily defeat session. These mice underwent autologous FMT via individual-matched transplantation of the fecal bacterial suspension prepared from their own pre-modeling (acclimation period) samples. The other CSDS group and the CON group received an equal volume of saline via gavage. Behavior assessments were conducted following the completion of the modeling period.

**Behavioral assessments**

Prior to behavioral assessment, mice exhibiting significantly low body weight or those that failed to compete successfully in social housing were excluded. This yielded final group sizes for behavioral analysis as follows: CON, n = 9; CSDS, n = 12; and CSDS+FMT, n = 11.

Depression-like phenotypes were evaluated using the Sucrose Preference Test (SPT) and Tail Suspension Test (TST). Anxiety-like behavior was assessed using the Open Field Test (OFT). Learning and memory function were evaluated using the Novel Object Recognition (NOR) test. All behavior tests, except the SPT, were conducted between 18:00 and 24:00. Before each test, mice were brought into the behavior testing room and allowed to acclimatize for at least 1 hour. Testing was performed in a quiet environment free from noise disturbances. The specific procedures were as follows:

OFT: Mice were placed individually in the center of a square arena (40 × 40 × 40 cm) and allowed to explore freely for 10 minutes. Their movement was tracked and recorded. Due to the innate aversion of rodents to open spaces, anxious mice tend to stay close to the walls, resulting in significantly reduced distance traveled in the central zone. In contrast, less anxious mice explore the center more freely. Therefore, the distance traveled in the central zone was used to quantify anxiety-like behavior.

SPT: This test was used to assess anhedonia, a core symptom of depression. Special drinking tubes (comprising a 15 mL centrifuge tube, a rubber stopper, and a ball-bearing sipper) were prepared, and mice were singly housed for the test. The procedure lasted four days. On day 1 (20:00), two bottles containing 1% sucrose solution were provided in symmetrical positions on the cage. On day 2 (20:00), one sucrose bottle was replaced with water. Twelve hours later (08:00 on day 3), the positions of the two bottles were switched to prevent side preference. On day 3 (20:00), food and water were removed. On the test day (day 4), pre-weighed bottles containing 1% sucrose solution and water were provided to each mouse at 20:00. The bottle positions were switched after 1 hour, and both bottles were removed and weighed at 22:00. Normal feeding was then resumed. The sucrose preference was calculated as: Sucrose Preference (%) = [Sucrose intake / (Sucrose intake + Water intake)] × 100%.

TST: This test assessed behavioral despair. The apparatus consisted of a box (45 cm high, 40 cm wide, 10 cm deep) with a horizontal bar (cross-section ~1 cm²) on top. The box was divided into three compartments by partitions to prevent visual contact between mice. A ~15 cm piece of medical PE tape was attached ~1 cm from the tip of the mouse's tail, and the mouse was suspended from the bar, with its head approximately 15 cm from the floor. The session was recorded for 6 minutes. The mouse was then removed, and the tape was carefully taken off. The apparatus was cleaned with ethanol and dried after each trial to eliminate olfactory cues. Immobility time during the final 4 minutes was analyzed.

NOR: This test evaluated learning and memory. The procedure spanned three days: Day 1 – habituation to the empty arena; Day 2 – familiarization with two identical objects; Day 3 – replacement of one familiar object with a novel object. Exploration time (sniffing within ~2 cm) of each object was recorded over a 5-minute session. A discrimination index was calculated to quantify recognition memory, with longer exploration of the novel object indicating better memory function.

**Definition of the depression-susceptible group**

Upon completion of the CSDS modeling, we subdivided the mice in the CSDS group (n=12) into susceptible (n=9) and resilient (n=3) subgroups. The classification was performed as follows: first, each of the four behavioral measures (OFT center time, SPT, TST, NOR) for CSDS mice was converted to a Z-score normalized to the control (CON) group. A composite depression-like phenotype score was then calculated using the formula: Composite Score = [ - (SPT Z) ] + [ - (Center Time Z) ] + [ (TST Z) ] + [ - (NOR Z) ]. A higher score indicates a more severe depression-like phenotype. The susceptible subgroup was defined as those mice with a composite score greater than the mean of the control group plus three standard deviations. Mice assigned to the resilient subgroup showed a composite score that was not statistically different from the control group but was significantly different from the susceptible subgroup.

**Tissue collection**

Following behavior tests, nine mice from each group were euthanized on ice. Trunk blood was collected into pre-chilled tubes. After clotting at room temperature for 30 minutes, samples were centrifuged at 3000 rpm for 15 minutes at 4°C. The supernatant (serum) was carefully aliquoted and rapidly frozen at -80°C. Brains were rapidly dissected on a chilled stage. The prefrontal cortex (PFC) and hippocampus (HIP) were isolated using a pre-cooled brain matrix, immediately flash-frozen in liquid nitrogen, and stored at -80°C. Fresh fecal pellets were collected directly from the rectum using sterile forceps, flash-frozen in liquid nitrogen, and stored at -80°C to preserve microbial DNA integrity. Serum and brain tissues were subsequently analyzed for energy metabolite levels via targeted metabolomics, and fecal samples were subjected to metagenomic sequencing, as described in Sections 2.4 and 2.5.

For the remaining three mice per group, deep anesthesia was induced using isoflurane. The thoracic cavity was opened to expose the heart. A blunted needle was inserted into the left ventricle, the right atrium was incised, and perfusion was initiated using a peristaltic pump. Mice were perfused sequentially with cold saline followed by ice-cold electron microscopy fixation buffer (2.5% glutaraldehyde and 2% paraformaldehyde in phosphate buffer). Perfused animals became rigid. The brain was quickly removed, and the PFC and HIP were dissected and placed in fresh fixative for several hours to overnight at 4°C for post-fixation.

**Electron microscopy sample preparation and observation**

Fixed tissue samples were washed thoroughly with phosphate buffer (3 × 10 min, or overnight; buffer was prepared fresh and stored at 4°C to prevent crystallization/contamination). Dehydration was performed using a graded ethanol series (50%, 70%, 80%, 90%, and 100% anhydrous ethanol; 10 min per step), followed by two 15-minute changes of 100% propylene oxide for resin infiltration. (Anhydrous sodium sulfate or copper sulfate was added to ethanol and propylene oxide to ensure anhydrous conditions).

Following dehydration, samples were infiltrated with epoxy resin mixtures (propylene oxide:resin at 1:1 and 1:3, then pure resin; 2 hours per step) in a drying oven with constant agitation. Samples were then embedded in molds and polymerized in an oven (35°C for 8 h, 45°C for 8 h, 60°C for 24 h). Polymerized blocks were trimmed for sectioning or stored in a desiccator.

Blocks were trimmed to a pyramidal tip (~1 × 1 mm) under a stereomicroscope. Ultrathin sections (~90 nm) were cut using an ultramicrotome and collected on copper grids. Sections were stained with 2% uranyl acetate (15 min, room temperature, sealed, dark) followed by Reynolds' lead citrate (2 min, room temperature, sealed, dark), then air-dried. Grids were examined using a transmission electron microscope. Target cells were located at low magnification (2,000–5,000X). High-magnification images were acquired (15,000–30,000X for overall mitochondrial morphology; >50,000X for cristae details). Key parameters assessed included overall morphology, distribution, and quantity.

**Mediation analysis**

The mediation analysis (mediation, dplyr, purr, tidyr packages) determines whether the mediating variable (M, energy metabolite levels) explains the relationship between the independent variable (X, relative abundance of gut microbiota) and dependent variable (Y, cognitive function). In this analysis, the relative abundance of gut microbiota was found to correlate with serum energy metabolite levels (pathway a), which are associated with cognitive function (pathway b). The total effect (c) of the gut microbiota on cognition is the association between the gut microbiota and cognition regardless of energy metabolite levels. The direct effect c' is the association between these variables while holding the energy metabolite variables constant. Indirect effects were derived by multiplying the a- and b-paths. When the direct effect is not significant (that is, c is significant but c' is not significant), the indirect effect completely mediates the total effect; whereas when the direct effect is significant (that is, the indirect effect and c' are significant), the partial mediates total effect. Confidence intervals for the indirect effects were estimated using 10000 bootstrap samples^3, 4^.

The code and parameters are as follows:

**# Load the necessary packages**

library(mediation)

library(dplyr)

library(purrr)

library(tidyr)

**# Read data**

data <- readxl::read_excel("129phe-meta-36micro.xlsx")

**# Define variable group**

bacteria_columns <- 2:37

metabolite_columns <- 38:51

phenotype_columns <- 52:53

bacteria_names <- names(data)[bacteria_columns]

metabolite_names <- names(data)[metabolite_columns]

phenotype_names <- names(data)[phenotype_columns]

**# Create an empty data frame to store the results**

results <- data.frame()

**# Batch mediation analysis function**

batch_mediation_analysis_v2 <- function(data, bacteria_names, metabolite_names, phenotype_names) {

results <- list()

df <- as.data.frame(data)

for(bacteria in bacteria_names) {

for(metabolite in metabolite_names) {

for(phenotype in phenotype_names) {

analysis_key <- paste(bacteria, metabolite, phenotype, sep = " -> ")

cat("Processing:", analysis_key, "\n")

if(!all(c(bacteria, metabolite, phenotype) %in% names(df))) {

cat("Skipping: variables not found\n")

next

}

tryCatch({

model_M <- do.call("lm", list(

as.formula(paste(metabolite, "~", bacteria)),

data = quote(df)

))

model_Y <- do.call("lm", list(

as.formula(paste(phenotype, "~", bacteria, "+", metabolite)),

data = quote(df)

))

cat(" Models fitted\n")

**# Conduct mediation analysis**

mediation_result <- do.call(mediation::mediate, list(

model.m = model_M,

model.y = model_Y,

treat = bacteria,

mediator = metabolite,

boot = TRUE,

sims = 10000

))

results[[analysis_key]] <- mediation_result

cat(" ✓ Success\n")

}, error = function(e) {

cat(" ✗ Error:", e$message, "\n")

})

}

}

}

return(results)

}

**# Carry out batch analysis**

system.time({

mediation_results <- batch_mediation_analysis_v2(data, bacteria_names, metabolite_names, phenotype_names)

})

**# A function for completely extracting the results of mediation analysis**

extract_complete_mediation_summary <- function(mediation_results) {

summary_list <- list()

for (i in seq_along(mediation_results)) {

pathway_name <- names(mediation_results)[i]

result <- mediation_results[[i]]

if (!is.null(result) && class(result) == "mediate") {

**# Extract the model coefficients and p-values**

model_M_summary <- summary(result$model.m)

model_Y_summary <- summary(result$model.y)

**# Extract the path a (independent variable → mediating variable) from model M**

a_coef <- coef(model_M_summary)[result$treat, "Estimate"]

a_pvalue <- coef(model_M_summary)[result$treat, "Pr(>|t|)"]

**# Extract the b path (mediator variable → dependent variable, controlling independent variable) from model Y**

b_coef <- coef(model_Y_summary)[result$mediator, "Estimate"]

b_pvalue <- coef(model_Y_summary)[result$mediator, "Pr(>|t|)"]

**# Extract the c' path from model Y (direct effect: independent variable → dependent variable, controlling for mediating variables)**

c_prime_coef <- coef(model_Y_summary)[result$treat, "Estimate"]

c_prime_pvalue <- coef(model_Y_summary)[result$treat, "Pr(>|t|)"]

**# Extract the total effect, direct effect, indirect effect, along with their confidence intervals and p-values**

summary_stats <- data.frame(

**# Path Identifier**

pathway = pathway_name,

bacteria = result$treat,

metabolite = result$mediator,

phenotype = as.character(formula(result$model.y))[2],

**# Path A: Independent variable → Mediating variable**

a_coefficient = a_coef,

a_pvalue = a_pvalue,

**# Path B: Mediator variable → Dependent variable**

b_coefficient = b_coef,

b_pvalue = b_pvalue,

**# Overall effect (c path)**

total_effect = result$tau.coef,

total_effect_pvalue = result$tau.p,

total_effect_CI_lower = result$tau.ci[1],

total_effect_CI_upper = result$tau.ci[2],

**# Direct Effect (Path c')**

direct_effect = result$z0,

direct_effect_pvalue = result$z0.p,

direct_effect_CI_lower = result$z0.ci[1],

direct_effect_CI_upper = result$z0.ci[2],

**# Indirect effect (mediating effect)**

indirect_effect = result$d0,

indirect_effect_pvalue = result$d0.p,

indirect_effect_CI_lower = result$d0.ci[1],

indirect_effect_CI_upper = result$d0.ci[2],

**# Intermediary ratio**

proportion_mediated = result$n0,

proportion_mediated_CI_lower = result$n0.ci[1],

proportion_mediated_CI_upper = result$n0.ci[2],

stringsAsFactors = FALSE

)

summary_list[[i]] <- summary_stats

} else {

**# For the analysis of failure, record the NA value.**

summary_list[[i]] <- data.frame(

pathway = pathway_name,

bacteria = NA, metabolite = NA, phenotype = NA,

a_coefficient = NA, a_pvalue = NA,

b_coefficient = NA, b_pvalue = NA,

total_effect = NA, total_effect_pvalue = NA,

total_effect_CI_lower = NA, total_effect_CI_upper = NA,

direct_effect = NA, direct_effect_pvalue = NA,

direct_effect_CI_lower = NA, direct_effect_CI_upper = NA,

indirect_effect = NA, indirect_effect_pvalue = NA,

indirect_effect_CI_lower = NA, indirect_effect_CI_upper = NA,

proportion_mediated = NA,

proportion_mediated_CI_lower = NA, proportion_mediated_CI_upper = NA,

stringsAsFactors = FALSE

)

}

}

return(do.call(rbind, summary_list))

}

**# Apply the extraction function**

mediation_complete_summary <- extract_complete_mediation_summary(mediation_results)

**# View the structure of the extracted results**

str(mediation_complete_summary)

head(mediation_complete_summary)

**# Save the complete result**

write.csv(mediation_complete_summary, "mediation_complete_summary.csv", row.names = FALSE)

**# Screen out the significant mediating effects (both the total effect and the indirect effect are significant)**

filter_significant_mediation <- function(mediation_summary,

total_effect_threshold = 0.05,

indirect_effect_threshold = 0.05) {

significant_results <- mediation_summary[

mediation_summary$total_effect_pvalue < total_effect_threshold &

mediation_summary$indirect_effect_pvalue < indirect_effect_threshold &

!is.na(mediation_summary$total_effect_pvalue) &

!is.na(mediation_summary$indirect_effect_pvalue),

]

**# Sort by the absolute value of the indirect effect**

significant_results <- significant_results[order(abs(significant_results$indirect_effect), decreasing = TRUE), ]

return(significant_results)

}

**# Application filtering**

significant_mediation <- filter_significant_mediation(mediation_complete_summary)

**# A function for adding significance markers to the p-value column**

add_significance_stars <- function(data) {

**# Copy the data frame and avoid modifying the original data**

result <- data

**# Identify all columns with p-values (column names containing "pvalue" or "pvalue")**

pvalue_cols <- grep("pvalue", names(result), ignore.case = TRUE, value = TRUE)

cat("找到的p值列:", pvalue_cols, "\n")

**# Add significance markers to each p-value column**

for (col in pvalue_cols) {

**# Create a new column name**

new_col_name <- paste0(col, "_sig")

**# Add significance markers based on the p-value**

result[[new_col_name]] <- sapply(result[[col]], function(p) {

if (is.na(p)) {

return("")

} else if (p < 0.001) {

return("***")

} else if (p < 0.01) {

return("**")

} else if (p < 0.05) {

return("*")

} else {

return("")

}

})

}

return(result)

}

**# Application function**

significant_mediation_with_stars <- add_significance_stars(significant_mediation)

**# Save the results with significant markings**

write.csv(significant_mediation_with_stars, "significant_mediation_with_stars.csv", row.names = FALSE)

**Random forest model**

To construct and evaluate the predictive model, the total cohort was first randomly divided into two independent subsets which comprised integrated microbiome and metabolomic features identified in the mediation analysis: a training set (n=90, 70% of the total cohort) and a testing set (n=39, 30% of the cohort). Based on the training set data, a random forest algorithm was employed to build an initial model (randomForest package). To identify the most predictive variables and prevent overfitting, the mean decrease in accuracy was calculated to assess the contribution of each feature to the model's predictive performance, thereby identifying key features. Subsequently, five-fold cross‑validation was applied within the training set to iteratively evaluate feature combinations, with the optimal feature subset determined by minimizing cross‑validation error. Using this selected optimal feature subset, the final model was run separately on the training set and the independent testing set, and receiver operating characteristic (ROC) curves were plotted. The area under the ROC curve (AUC) was calculated to quantitatively evaluate the model's discriminative ability (pROC package).

The code and parameters are as follows:

**# Construct a function**

rfcv1 <-function (trainx, trainy, cv.fold = 5, scale = "log", step = 0.5, mtry = function(p) max(1, floor(sqrt(p))), recursive = FALSE, ...)

{

classRF <- is.factor(trainy)

n <- nrow(trainx)

p <- ncol(trainx)

if (scale == "log") {

k <- floor(log(p, base = 1/step))

n.var <- round(p * step^(0:(k - 1)))

same <- diff(n.var) == 0

if (any(same))

n.var <- n.var[-which(same)]

if (!1 %in% n.var)

n.var <- c(n.var, 1)

}

else {

n.var <- seq(from = p, to = 1, by = step)

}

k <- length(n.var)

cv.pred <- vector(k, mode = "list")

for (i in 1:k) cv.pred[[i]] <- rep(0,length(trainy))

if (classRF) {

f <- trainy

}

else {

f <- factor(rep(1:5, length = length(trainy))[order(order(trainy))])

}

nlvl <- table(f)

idx <- numeric(n)

for (i in 1:length(nlvl)) {

idx[which(f == levels(f)[i])] <- sample(rep(1:cv.fold,

length = nlvl[i]))

}

res=list()

for (i in 1:cv.fold) {

all.rf <- randomForest(trainx[idx != i, , drop = FALSE],

trainy[idx != i],importance = TRUE)

aa = predict(all.rf,trainx[idx == i, , drop = FALSE],type="prob")

cv.pred[[1]][idx == i] <- as.numeric(aa[,2])

impvar <- (1:p)[order(all.rf$importance[, 3], decreasing = TRUE)]

res[[i]]=impvar

for (j in 2:k) {

imp.idx <- impvar[1:n.var[j]]

sub.rf <- randomForest(trainx[idx != i, imp.idx,

drop = FALSE], trainy[idx != i]

)

bb <- predict(sub.rf,trainx[idx ==i,imp.idx, drop = FALSE],type="prob")

cv.pred[[j]][idx == i] <- as.numeric(bb[,2])

if (recursive) {

impvar <- (1:length(imp.idx))[order(sub.rf$importance[,

3], decreasing = TRUE)]

}

NULL

}

NULL}

if (classRF) {

error.cv <- sapply(cv.pred, function(x) mean(factor(ifelse(x>0.5,1,0))!=trainy))

}

else{

error.cv <- sapply(cv.pred, function(x) mean((trainy -

x)^2))

}

names(error.cv) <- names(cv.pred) <- n.var

list(n.var = n.var, error.cv = error.cv, predicted = cv.pred,res=res)

}

**## Load the necessary packages ##**

set.seed(1234)

library(randomForest)

library(pROC)

**## read the metabolic data and the OTU data ##**

data <- read.csv("meta-micro.csv", header = T)

index <- sample(2,nrow(data),replace = TRUE,prob=c(0.7,0.3))

train <- data[index==1,]

test <- data[index==2,]

**## Convert the group into a factor**

train$group<-as.factor(train$group)

**######5*10_crossvalidation####**

**##source("ramdomforest.crossvalidation.r") ## Apply the rfcv1 function**

result <- replicate(5, rfcv1(train[,-ncol(train)], train$group, cv.fold=10,step=0.9), simplify=FALSE)

error.cv <- sapply(result, "[[", "error.cv")

matplot(result[[1]]$n.var, cbind(rowMeans(error.cv), error.cv), type="l", lwd=c(2, rep(1, ncol(error.cv))), col=1, lty=1, log="x", testlab="Number of variables", ylab="CV Error")

abline(v=7,col="pink",lwd=2)

error.cv.cbm<-cbind(rowMeans(error.cv), error.cv)

cutoff<-min (error.cv.cbm[,1])+sd(error.cv.cbm[,1])

error.cv.cbm[error.cv.cbm[,1]<cutoff,]**## Derive the index of the minimum error rate**

**#####pick 10 marker by corossvalidation#######**

k=1

**# Set the number of columns and match it with your own data.**

b <- matrix(0,ncol=106,nrow=50)

for(i in 1:5){

for(j in 1:10){

b[k,]<-result[[i]]$res[[j]]

k=k+1 } }

mlg.list<-b[,1:10]

list<-c()

k=1

for(i in 1:10){

for(j in 1:50){

list[k]<-mlg.list[j,i]

k=k+1 } }

mlg.sort<-as.matrix(table(list)) **## Sort**

mlg.sort<-mlg.sort[rev(order(mlg.sort[,1])),]

pick<- as.numeric(names(head(mlg.sort,7)))

tmp=train[,-ncol(train)]

mlg.pick<-colnames(tmp)[pick]

write.table(mlg.pick,"crossvalidation.ttestt", sep="\t",quote=F)

**###train.set**

**#train1 <- train[,c(pick,127)]**

set.seed(1234)

train1 <-data.frame(train)

train1.rf <- randomForest(group ~ Isocitric.acid

+ Adenine

+ Cyclic.AMP

+ AMP

+ Ornithine

+ L.Cystine

+ L.citrulline

, data =train, importance = TRUE)**## By using random forest, examine the first n markers in the training set**

varImpPlot(train1.rf)

train1.pre <- predict(train1.rf,type="prob")

p.train<-train1.pre[,2]

boxplot(p.train~train$group,col=c(3,4),main="Probability of depression")

write.table(p.train,"predict.in.train.ttestt", sep="\t",quote=F)

**########ROC in train######**

library(pROC)

roc(train$group,p.train)

roc1 <- roc(train$group, p.train, percent=TRUE, partial.auc.correct=TRUE, ci=TRUE, boot.n=100, ci.alpha=0.9, stratified=FALSE, plot=F, auc.polygon=TRUE, matest.auc.polygon=TRUE, grid=TRUE )

roc1 <- roc(train$group, p.train, ci=TRUE, boot.n=100, ci.alpha=0.9, stratified=FALSE, plot=TRUE, percent=roc1$percent,col=2)

sens.ci <- ci.se(roc1, specificities=seq(0, 100, 5))

plot(sens.ci, type="shape", col=rgb(0,1,0,alpha=0.2))

plot(sens.ci, type="bars")

plot(roc1,col=2,add=T)

legend("bottomright",c(paste("AUC=",round(roc1$ci[2],2),"%"), paste("95% CI:",round(roc1$ci[1],2),"%-",round(roc1$ci[3],2),"%")))

**##end##**

**##########test.set###########**

set.seed(999)

predict(train1.rf, test)

set.seed(999)

test1.pre<-predict(train1.rf, test,type="prob")

p.test<-test1.pre[,2]

boxplot(p.test~test$group,col=c(3,4),main="Probability of schizophrenia")

write.table(p.test,"predict.in.test.ttestt", sep="\t",quote=F)

**########ROC in test######**

roc(test$group,p.test)

roc1 <- roc(test$group, p.test, percent=TRUE, partial.auc.correct=TRUE, ci=TRUE, boot.n=100, ci.alpha=0.9, stratified=FALSE, plot=F, auc.polygon=TRUE, matest.auc.polygon=TRUE, grid=TRUE )

roc1 <- roc(test$group, p.test, ci=TRUE, boot.n=100, ci.alpha=0.9, stratified=FALSE, plot=TRUE, percent=roc1$percent,col=2)

sens.ci <- ci.se(roc1, specificities=seq(0, 100, 5))

plot(sens.ci, type="shape", col=rgb(0,1,0,alpha=0.2))

plot(sens.ci, type="bars")

plot(roc1,col=2,add=T)

legend("bottomright",c(paste("AUC=",round(roc1$ci[2],2),"%"), paste("95% CI:",round(roc1$ci[1],2),"%-",round(roc1$ci[3],2),"%")))

**##end##**

**Supplementary tables**

**table S1.** Intergroup comparison of metabolites associated with energy metabolism

**table S2.** Different gut bacterial species between groups

**table S3.** LEfSe analysis results of different bacteria between groups

**table S4.** GMM modules with significant differences between groups

**table S5.** Species involved in energy metabolic pathways obtained based on HUMAnN3 analysis

**table S6.** Enzymes associated with energy metabolism produced by the gut microbiota

**table S7.** Relative abundance of gut microbiota species associated with four enzymatic reactions in energy metabolic pathways

**table S8.** Mediation analysis results for cognitive function and symptoms

**table S9.** The gut bacteria phylum with differences in the three comparison groups

**table S10.** The gut bacteria species with differences in the three comparison groups

**table S11.** The KEGG pathway based on gut microbiota with differences in the three comparison groups

**table S12.** Comparison of energy metabolites in serum, PFC and HIP among the three groups

**Supplementary figures**

**Figure S1.** The correlation between self-rating scales and clinician-rated scales in depression.

**Figure S2.** The association between differential energy metabolites and their enrichment directions across different groups.

**Figure S3.** Associations between energy metabolism-related metabolites and thyroid hormone profiles.

**Figure S4.** Significantly dysregulated energy metabolism pathways annotated by HUMAnN3 metagenomic analysis.

**Figure S5.** Differential abundance of gut microbial species participating in mitochondrial fatty acid biosynthesis initiation pathway (PWY66-429).

**Figure S6.** Associations between inter-group 92 differential gut microbial species and energy metabolites, blood lipids, and immune cells.

**Figure S7.** (A) Intergroup differences in expression levels of energy metabolism enzymes. (B-C) Relative abundance of *Klebsiella_pneumoniae* and *Escherichia_coli* associated with four enzymatic reactions in energy metabolic pathways.

**Figure S8.** Energy metabolites mediate the regulatory effect of gut microbiota on cognitive function: (A) Schematic model of energy metabolites mediating gut microbiota-cognition relationships. Path a: Association between gut microbial species and energy metabolites. Path b: Association between energy metabolites and cognitive performance. Total effect c: Overall gut microbiota-cognition relationship. Direct effect c': Microbiota-cognition link independent of metabolites. Indirect effect ab: Mediated pathway through energy metabolites. (B-E) Energy metabolites mediated relationships between gut microbiota and objective cognitive tests, including Choice Reaction Time (CRT), 1-back Working Memory Task (1-back), Digit Symbol Substitution Test (DSST), and composite objective cognitive score. (F) Mediation relationship of gut microbiota and subjective cognition (PDQ-5-D). (G) Mediation relationship of gut microbiota and total Thinc-it scores.

**Figure S9.** Random forest-based diagnostic model: (A) Feature selection through five iterations of 10-fold cross-validation to optimize biomarker panel based on minimal cross-validation error. (B-C) Receiver operating characteristic (ROC) curves with corresponding area under the curve (AUC) values in training and validation cohorts, respectively. (D) Variable importance plot displaying diagnostic biomarkers ranked by Mean Decrease Accuracy.

**Figure S10.** Behavioral phenotype analysis of mice from different treatment groups.

(A-D) show the results of the open field test (OFT), sucrose preference test (SPT), novel object recognition (NOR) test, and tail suspension test (TST), respectively. CSDS_susceptible subgroup: mice were classified as susceptible if their composite depression-like phenotype score was greater than the mean of the CON group plus three standard deviations. ****P* < 0.001, ***P* < 0.01, **P* < 0.05.

**Figure S11.** Comparative analysis of gut microbiota differences and effect sizes of key enzymes across groups. (A) LEfSe analysis of gut microbiota differences between the CSDS and CON groups: The horizontal axis represents the Linear Discriminant Analysis (LDA) score, and the vertical axis lists the differentially abundant taxa at different classification levels (from phylum to species, with level prefixes indicated: p__, phylum; c__, class; o__, order; f__, family; g__, genus; s__, species). (B) LEfSe analysis of gut microbiota differences between the CSDS-FMT and CSDS groups. (C) LEfSe analysis of gut microbiota differences between the CON and CSDS-FMT groups. For all LEfSe analyses, the screening criteria for differentially abundant taxa were an LDA score > 2 and a *P*-value < 0.05. (D) Forest plot of effect sizes for core metabolic enzymes. The horizontal axis shows the Mean Difference, and the vertical axis lists five key metabolic enzymes. The plot compares the differences in enzyme expression across three group comparisons: CSDS vs. CON, CSDS-FMT vs. CSDS, and CSDS-FMT vs. CON.

References

(1) Huang, Z.; Chen, J.; Li, B.; Zeng, B.; Chou, C. H.; Zheng, X.; Xie, J.; Li, H.; Hao, Y.; Chen, G.; et al. Faecal microbiota transplantation from metabolically compromised human donors accelerates osteoarthritis in mice. *Ann Rheum Dis* **2020**, *79* (5), 646–656. DOI: 10.1136/annrheumdis-2019-216471.

(2) Gao, Y.; Peng, K.; Bai, D.; Bai, X. Y.; Bi, Y.; Chen, A.; Chen, B.; Chen, F.; Chen, J.; Chen, L.; et al. The Microbiome Protocols eBook initiative: Building a bridge to microbiome research. *Imeta* **2024**, *3* (2), e182. DOI: 10.1002/imt2.182.

(3) Preacher, K. J.; Hayes, A. F. Asymptotic and resampling strategies for assessing and comparing indirect effects in multiple mediator models. *Behav Res Methods* **2008**, *40* (3), 879–891. DOI: 10.3758/brm.40.3.879.

(4) Baron, R. M.; Kenny, D. A. The moderator-mediator variable distinction in social psychological research: conceptual, strategic, and statistical considerations. *J Pers Soc Psychol* **1986**, *51* (6), 1173–1182. DOI: 10.1037//0022-3514.51.6.1173.
